# Aggregation Behavior and Application Properties of Novel Glycosylamide Quaternary Ammonium Salts in Aqueous Solution

**DOI:** 10.3390/molecules29122749

**Published:** 2024-06-09

**Authors:** Yunkai Wang, Zeyu Chen, Erzhuang Zhang, Lifei Zhi, Martino Di Serio, Guoyong Wang, Yan Wang, Xiaoming Li, Xudong Liu, Ying Huang

**Affiliations:** 1 College of Chemical Engineering and Technology, Taiyuan University of Science and Technology, Taiyuan 030024, China; wyk990306@163.com (Y.W.); czy15535371231@163.com (Z.C.); aa1162791419@163.com (E.Z.); wanggy@tyust.edu.cn (G.W.); kdwyan@126.com (Y.W.); lxmlily2014@126.com (X.L.); 2 Department of Chemical Sciences, University of Naples Federico II, 80138 Napoli, Italy; martion.diserion@gmail.com; 3 Research Institute of Livestock and Aquatic Product Inspection, Shanxi Inspection and Testing Center, Taiyuan 030006, China; 13835179079@163.com; 4 Taiyuan Hengdeyuan Animal Protection Technology Development Co., Ltd., Taiyuan 030003, China; huangying0351@163.com; 5 Shanxi Livestock and Poultry Breeding Co., Ltd., Jinzhong 031800, China

**Keywords:** surfactant, lactose amide, quaternary ammonium salts, surface activity, vesicle

## Abstract

Amidation of lactobionic acid with N,N-dimethylaminopropyltriamine was conducted to obtain N-(3′-dimethylaminopropyl)-lactamido-3-aminopropane (DDLPD), which was quaternized with bromoalkanes of different carbon chain lengths to synthesize double-stranded lactosylamide quaternary ammonium salt N-[N′[3-(lactosylamide)]propyl-N′-alkyl] propyl-N,N-dimethyl-N-alkylammonium bromide (C_n_DDLPB, n = 8, 10, 12, 14, 16). The surface activity and the adsorption and aggregation behaviors of the surfactants were investigated via equilibrium surface tension, dynamic light scattering, and cryo-electron microscopy measurements in an aqueous solution. The application properties of the products in terms of wettability, emulsification, foam properties, antistatic, salt resistance, and bacteriostatic properties were tested. C_n_DDLPB exhibited a low equilibrium surface tension of 27.82 mN/m. With an increase in the carbon chain length, the critical micellar concentration of C_n_DDLPBD decreased. Cryo-electron microscopy revealed that all products except C_8_DDLPB formed stable monolayer, multi-layer, and multi-compartmental vesicle structures in an aqueous solution. C_14_DDLPB has the best emulsification performance on soybean oil, with a time of 16.6 min; C_14_DDLPB has good wetting and spreading properties on polytetrafluoroethylene (PTFE) when the length of carbon chain is from 8 to 14, and the contact angle can be lowered to 33°~40°; C_n_DDLPB has low foam, which is typical of low-foaming products; C_8_DDLPB and C_10_DDLPB both show good antistatic properties. C_8_DDLPB and C_14_DDLPB have good salt resistance, and C_12_DDLPB has the best antimicrobial property, with the inhibition rate of 99.29% and 95.28% for E. coli and Gluconococcus aureus, respectively, at a concentration of 350 ppm.

## 1. Introduction

Among global environmental issues, carbon emissions have become a key issue that has received a lot of attention [1,2]. Of particular note is China’s high level of carbon emissions between 2009 and 2022 [3]. To address this challenge, China made a solemn commitment at the 75th session of the United Nations General Assembly to adopt dual carbon targets, i.e., carbon peaking and carbon neutrality, as a part of its core carbon reduction strategy. Owing to their green, environmentally friendly, and resource-saving characteristics, biobased chemicals are in alignment with the “dual-carbon” policy development; these chemicals are gradually becoming a new leading industry in the contemporary world of scientific and technological innovation and economic development. China’s “13th Five-Year Plan” and “14th Five-Year Plan” also clearly reveal that focus will be placed on the development of biobased industries [4]. Biobased chemicals, which are derived from renewable resources, are bulk chemicals and fine chemicals prepared from biomass as raw materials, exhibiting several advantages such as carbon reduction and sustainability [5,6,7]. Biobased chemicals produce very little waste during their production process, and most of this waste can be recycled due to their green and healthy characteristics. Their easily degradable nature also provides an effective way to solve the pollution problem of petroleum-based plastics [8,9]. Sugars are typical representatives of biomass raw materials and prepared from natural renewable resources such as starch, exhibiting not only abundant sources but also cost-effectiveness [10]. The use of sugar groups as hydrophilic groups in surfactants endows them with several advantages: (1) Sugar groups endow surfactants with considerably good biodegradation performance and toxicological properties. Moreover, they are mild and non-irritating to the skin and eyes as well as easily biodegradable under anaerobic and aerobic conditions [11,12,13]. (2) Sugar groups contain multiple hydroxyl groups, the oleophobicity and hydrophilicity of which is greater than those of conventional polyoxyethylene ether surfactants; the oleophobicity of a sec-hydroxyl group is 4.5 times greater than that of polyethylene oxide. In oil–water systems, sugar-based surfactants exhibit better interfacial chemistry [14]. (3) Sugar groups exhibit higher resistance to hard water because they can form complexes with metal ions such as Ca^2+^ to form water-soluble complex ions. (4) Sugar groups also endow surfactants with insecticidal and herbicidal or antimicrobial activity [15,16,17]. Introducing sugar groups into the molecular structure of cationic surfactants may improve their irritation, toxicity, biodegradability, water solubility, compatibility, etc. [18]. Various cationic surfactants derived from sugar groups exhibit advantages, including green characteristics, natural and renewable raw materials, facile biodegradability, and multifunctionality; these surfactants are also safe and mild for human body. Therefore, these surfactants have globally attracted considerable research attention [19,20,21].

**Figure 1:**
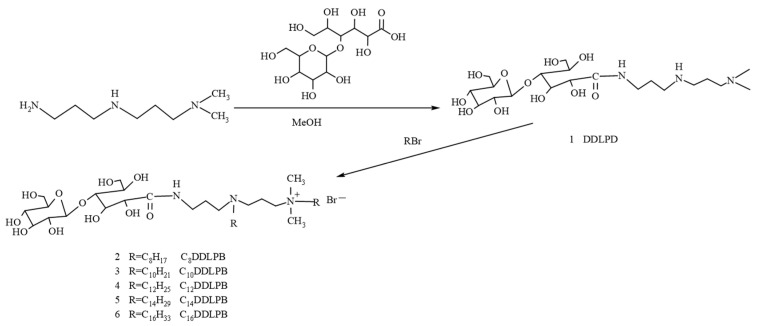
Synthesis roadmap of double-chain lactosamine quaternary ammonium salts (C_n_DDLPB).

Herein, a double-chain lactide amide quaternary surfactant (C_n_DDLPB), which combines the advantages of a sugar group, an amide bond, and a cationic surfactant, was synthesized via the amine ester reaction of lactobionic acid with an alkyl amine and then quaternized with bromoalkanes (the reaction and synthesis routes are shown in Figure 1), and the structures of the intermediates and products were characterized via Fourier transform infrared (IR) spectroscopy, nuclear magnetic resonance (NMR) spectroscopy, and mass spectrometry(MS). In addition, aggregation behavior and application properties of C_n_DDLPB were also comprehensively investigated.

## 2. Results and Discussion

### 2.1. Structure Identification

The chemical structures of the raw materials, synthesized intermediates, and target compounds were characterized using FTIR, ^1^H NMR, and ^13^C NMR spectroscopies as well as other characterization methods. The spectra of all products are in the Supporting Information.

Figure S1a shows the FTIR spectrum of N,N-dimethyldipropylenetriamine. The peak observed at 2931 cm^−1^ corresponds to the symmetrical telescopic vibration of C–H of the methyl group, while the peak observed at 2762 cm^−1^ corresponds to the symmetrical telescopic vibration of C–H of the methylene group [22]. Figure S1b shows the FTIR spectrum of lactose. A broad and strong absorption peak observed at 3400–3200 cm^−1^ corresponds to the stretching vibration of the O–H group in lactobionic acid, the absorption peak observed at 1730 cm^−1^ corresponds to the stretching vibration of the C=O bond in lactobionic acid, and the strong peak observed at 1030 cm^−1^ corresponds to the C–O bond stretching vibration.

In the spectrum of DDLPD in Figure S1c, absorption peaks corresponding to the O–H group in lactobionic acid and C–H of N, N-dimethyldipropenyltriamine are observed; the two strong characteristic absorption peaks observed at 1650 and 1540 cm^−1^ correspond to the telescopic vibration of the C=O bond in amide groups and the bending vibration of N–H, respectively, indicating that amide bonds are generated.

### 2.2. Electrospray Mass Spectrometry (EMS)

The synthesized glycosylamide quaternary ammonium salt was analyzed via ESI-MS, and its composition was analyzed and identified according to its molecular weight: the relative molecular mass of the expected product C_12_DDLPB is 916. Figure S16 shows the ESI-MS spectrum of the target product C_12_DDLPB. The quaternary ammonium surfactant C_12_DDLPB exhibits an (M–H)^+^ quasimolecular ion peak at 915.35 (*m*/*z*). The quasimolecular ion is relatively stable after losing one electron; Br^−^ is bombarded using the positive-ion mode, and a strong [M–Br]^+^ fragmentation peak is observed at *m*/*z* 836.86. The fragmentation peak of [M-Br- OH-OH]^+^ is observed at *m*/*z* 746.88, the fragmentation peak of [M-Br-CH_3_O-C_2_H_5_O_2_-O-O]^+^ is observed at *m*/*z* 612.55, and the fragmentation peak of [M-Br-CH_3_O-C_2_H_5_O_2_-OH-OH -H-H]^+^ is observed at *m*/*z* 608.85. The *m*/*z* values of the molecular ion peaks of C_8_DDLPB, C_10_DDLPB, C_14_DDLPB, and C_16_DDLPB are 803.49, 862.51, 974.38, and 1029.45, respectively, which are consistent with the expected molecular masses of the products (Supplementary Material Figures S14–S18) [23].

The combined FTIR spectroscopy, ^1^H NMR spectroscopy, ^13^CNMR spectroscopy, and ESI-MS results reveal that the double-stranded lactose amide quaternary surfactants are successfully synthesized.

### 2.3. Surface Tension

**Figure 2:**
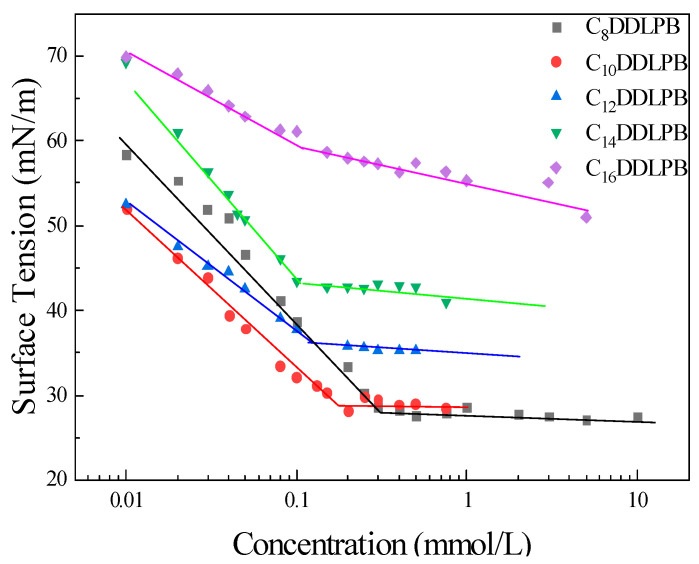
Surface tension of C_n_DDLPB in an aqueous solution as a function of concentration.

The surface tension of C_n_DDLPB was determined using the hanging drop method at 25 °C ± 0.1 °C. The surfactant solution was prepared from RO water; the surface tension of RO water was 72 ± 0.3 mN/m. The surface tension versus concentration curve reveals an inflection point in the concentration, corresponding to the critical micellar concentration (CMC). Before the turning point is reached (Figure 2), a linear relationship between the increase in the surfactant concentration and the decrease in surface tension is observed, indicating that the surfactant molecules begin to arrange closely at the interface. After reaching the CMC, the surface tension remains constant.

According to Equations (1)–(3) [24,25], the amount of adsorption at saturation at the gas/liquid interface (Γ_max_), cross-sectional area per molecule A_min_, and surface activity efficiency pC_20_ can be estimated:

(1) $$\Gamma_{\max}=-\frac{1}{2.303\mathrm{nRT}}{\left(\frac{\partial \gamma }{\partial \mathrm{lgc}}\right)}_{T}$$

(2) $$A_{\min}=\frac{10^{16}}{N_{A}\Gamma_{\max}}$$

(3) $${\mathrm{pC}}_{20}=-{\mathrm{logC}}_{20}$$

where γ represents the surface tension, mN/m; T represents the absolute temperature, K; and R represents the gas constant, 8.314 J/(mol·K). Moreover, the value of n for an ionic surfactant type 1-1 is considered to be 2, N_A_ represents the Avogadro constant, and C_20_ represents the concentration of the surfactant required to reduce the surface tension of water by 20 mN/m.

CMC/C_20_ indicates the difficulty and simplicity of the surfactant adsorption and micellization process. The higher CMC/C_20_, the easier the tendency for the surfactant to adsorb on the interface than that to form a micelle.

**Figure 3:**
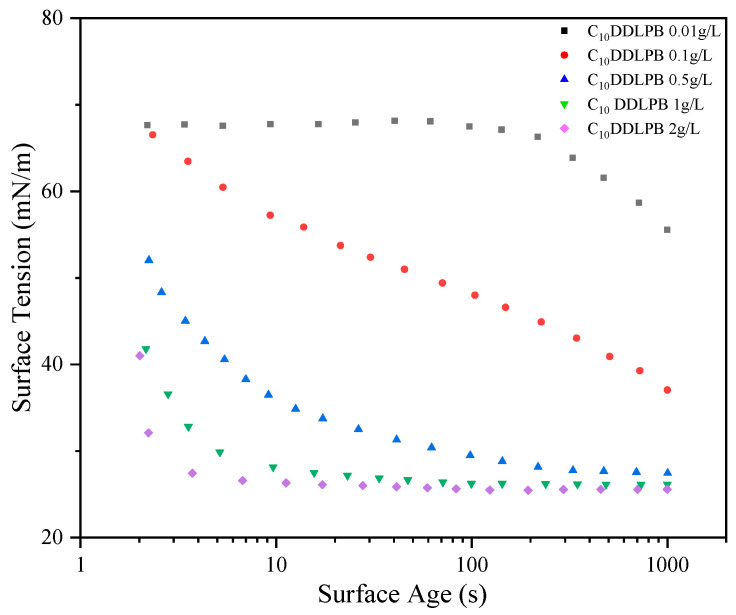
Dynamic surface tensions vs. surface age for 0.01 g/L–2 g/L C_n_DDDLPB.

The CMC values of the five double-chain lactose amide quaternary ammonium salts (C_n_DDLPB) decrease in the order of C_8_DDLPB > C_10_DDLPB > C_12_DDLPB > C_14_DDLPB > C_16_DDLPB (Figure 3). Their CMC values decrease linearly with the growth of the hydrophobic carbon chain [26]. This result is attributed to the fact that, when two carbon chains of the surfactant molecule become longer, the space occupied by the molecules arranged at the interface is saturated more rapidly. The larger the space occupied by the two carbon chains of the surfactant molecules, the faster the molecules arranged at the interface reach saturation; at the same time, the longer the carbon chains, the hydrophobicity of the hydrocarbon bonds is gradually enhanced, indicating that the separation formed between the hydrophilic head groups under the action of electrostatic repulsive force is hindered, rendering a significant promotion effect on the development of aggregates in the surfactants. The shorter the carbon chain, the lower the surface tension. The surface tension of the C_8_DDLPB aqueous solution can be reduced to 27.82 mN/m. The inflection point of CMC on the curve of the change in the surface tension with the C_16_DDLPB concentration is not extremely clear, which may be attributed to the fact that the two hydrophobic carbon chains are extremely long and lead to poor solubility and a low surface activity.

With an increase in the length of the carbon chain, Г_max_ decreases (Table 1). When the carbon chain becomes longer, the obstruction of the molecules to occupy a position in space increases. The possibility of the dense arrangement of molecules within the unit surface decreases, and the cross-sectional area Amin per molecule increases. The pC_20_ value of C_10_DDLPB is the highest, indicative of the higher adsorption efficiency at the interface.

### 2.4. Dynamic Surface Tension

Dynamic surface tension can be used to study the adsorption–diffusion kinetics of surfactants at the air–water interface. In this study, the dynamic surface tension changes in C_10_DDLPB were measured in the concentration range of 0.01 g/L–2 g/L by the suspended droplet method, and the dynamic surface tension of C_n_DDLPB with five different carbon chain lengths at a concentration of 1 g/L (25 °C) was measured in detail.

Figure 3 demonstrates the dynamic surface tension curves of C_10_DDLPB at different concentrations, from which it can be seen that the surface activity increases with increasing concentration, and the higher the concentration, the faster the adsorption rate.

**Figure 4:**
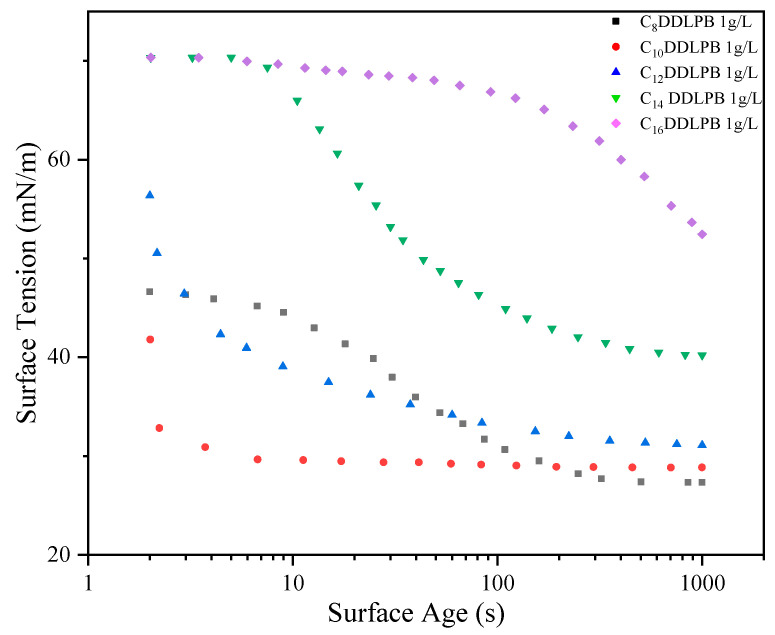
Dynamic surface tensions vs. surface age for 1 g/L C_n_DDDLPB.

Figure 4 demonstrates the surface tension versus surface age plots of C_n_DDLPB with different carbon chain lengths at 1 g/L concentration. With the increase in surface age, the surface tension of each surfactant gradually decreased and finally reached a stable value. This indicates that the C_n_DDLPB molecules undergo the process of diffusion–adsorption, reaching a steady state at the liquid surface. The ability to reduce the surface tension has a tendency to increase with the decrease in carbon chain length, which may be related to the fact that the shorter carbon chain molecules are easier to be arranged and form a compact structure at the interface. However, it is noteworthy that the adsorption rates of the surfactants increased with decreasing carbon chain length, except for C_8_DDLPB, for which this anomaly may be attributed to the shorter hydrophobic chain.

### 2.5. Aggregation Behavior in Aqueous Solutions

**Figure 5:**
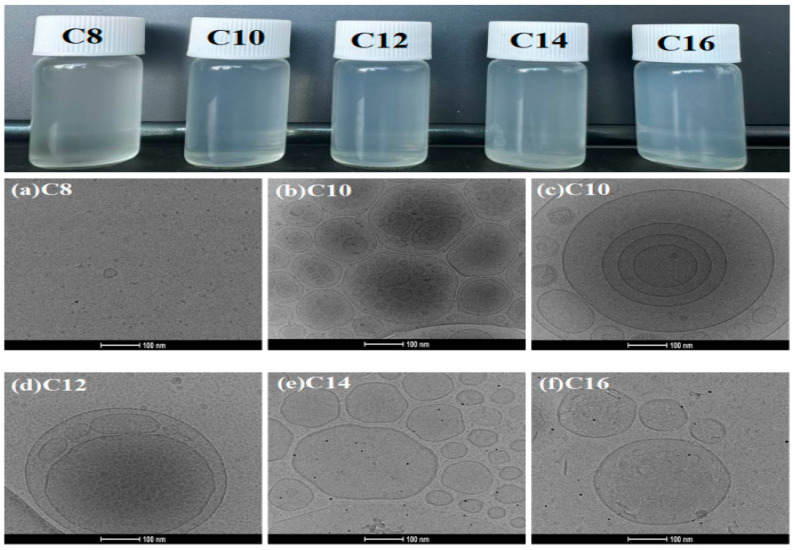
Aggregation behavior of C_n_DDLPB under cryo-electron microscopy.

The aggregation behavior of C_n_DDLPB at a concentration of 5 g/L in aqueous solution was observed using cryo-transmission electron microscopy (Cryo-TEM) at a magnification of 92,000 times, as shown in Figure 5. Except for C_8_DDLPB, which forms ordinary micelles with a diameter of approximately 20 nm in an aqueous solution, the other samples form single-layered, multi-layered, or multi-compartmental vesicle structures, with vesicle diameters ranging from approximately 100 to 400 nm. Among them, C_10_DDLPB and C_12_DDLPB formed multi-compartmental vesicles, and C_14_DDLPB and C_16_DDLPB formed single-layer vesicle structures. Figure 5b,c and Supporting Information Figure S19b,c show the cryogenic transmission electron microscopy images of C_10_DDLPB samples kept for 1 month and 24 h, respectively. The magnification of the cryo-electron microscope in the Supporting Information is 45,000×. With an increase in the time of placing the samples, more vesicles are formed, indicating that the vesicle-forming system is more stable with an increase in the sample storage time and that more vesicles are aggregated to form a multi-compartmental vesicle structure. The results reveal that the vesicle-forming system is more stable with an increase in the sample storage time.

The appearance of the prepared solutions also reveals that C_8_DDLPB is a translucent light gray solution and that C_10_DDLPB-C_16_DDLPB is a light blue solution. During vesicle formation, surfactant molecules aggregate at the water–oil interface and form a closed structure with hydrophobic chains inside and hydrophilic chains outside. The length of the hydrophobic chains is critical to the stability of the vesicles. Surfactant molecular structures with two hydrocarbon chains and large head groups are prone to spontaneous vesicle formation [27,28,29]. The molecular structure of the surfactant synthesized herein (C_n_DDLPB) is in agreement with such features. However, in case of a short-carbon chain, sufficient hydrophobic forces cannot be provided to maintain the stability of vesicles, resulting in the facile rupture of vesicles. In addition, surfactant molecules with a short-carbon chains exhibit a high degree of expansion in an aqueous solution, indicating that the formation of tight aggregation structures by these short-carbon chains is difficult and that water molecules cannot be wrapped effectively to form complete vesicle structures.

**Figure 6:**
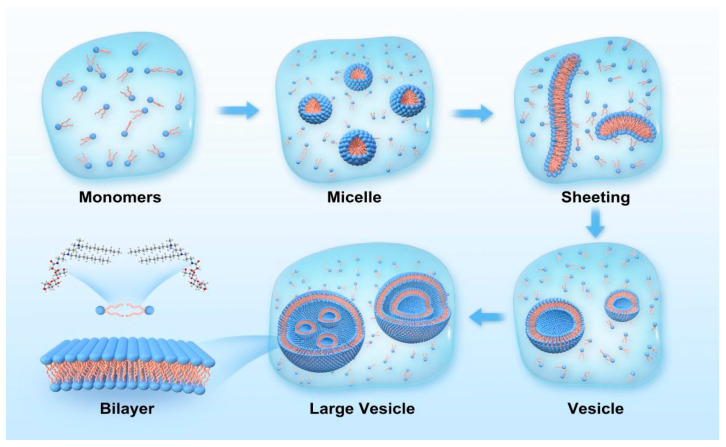
Schematic diagram of the vesicle formation of C_n_DDLPB.

The mechanism of C_n_DDLPB solution vesicle formation is shown in Figure 6: (1) First, C_n_DDLPB surfactant molecules are dissolved in water, and the hydrophilic head interacts with water molecules and is surrounded by them, which are dispersed in solution as monomers. (2) Formation of micelles: at the water/oil interface or water/air interface, the hydrophobic tails of the surfactant molecules interact with the oil or air phase, leading to the adsorption of the molecules on the interface. Formation of a stable interface: when the solution concentration reaches the CMC, the active molecules form monolayer micelles [30]. (3) Formation of flexible bilayers: With an increase in the concentration, the micelles begin to disperse and reorganize into flexible bilayer structures. With an increase in the size of the molecular films, they begin to inwardly bend spontaneously to form curved structures to reduce edge energy; this closure can be facilitated by hydrophobic forces between the hydrophobic tails [31,32]. (4) Unstable vesicles: when the bilayer is completely closed, small unstable vesicles are formed [33]. (5) Formation of stable large vesicles: Over time, multiple small vesicles merge with each other to form a stable large vesicle [34,35]. The ultimate stability of a vesicle depends on the balance between hydrophobic effects and surface tension. The stronger the interaction force of the hydrophobic tail, the higher the stability of the vesicle. At the same time, the film formed by surfactant molecules at the interface can also reduce the surface energy of the system and make the vesicle structure more stable. C_10_DDLPB and C_12_DDLPB exhibit the best balance of the hydrophobic effect and surface tension, which are more likely to aggregate on the membrane surface and induce the neighboring layers to come closer together, leading to the gradual stacking of the membrane layers to form multi-layer and multi-compartmental structures.

Most cationic surfactants cannot spontaneously form vesicles. Therefore, C_n_DDLPB with a long-carbon chain demonstrates good application prospects in slow-release drug carriers, template agents, biofilm mimicry, microreactors, and the cosmetic and food industries.

**Figure 7:**
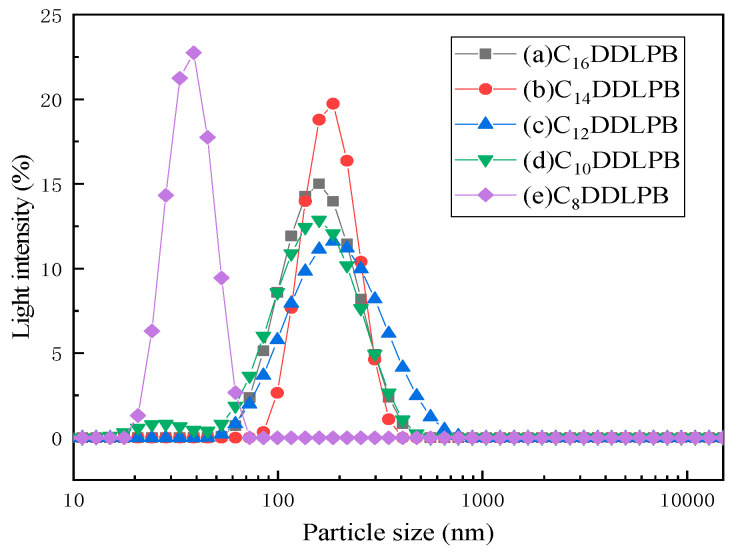
Particle size distributions of (**a**) C_16_DDLPB, (**b**) C_14_DDLPB, (**c**) C_12_DDLPB, (**d**) C_10_DDLPB, and (**e**) C_8_DDLPB.

Figure 7 shows the particle size distribution of the 5 g/L C_n_DDLPB sample. C_n_DDLPB exhibits a single-peak particle distribution, and the overall particle sizes range from 20 nm to 1000 nm. The particle sizes of C_16_DDLPB and C_10_DDLPB exhibit a narrower distribution between 50 nm and 400 nm (Figure 7a,d), indicating that the diameters of the formed aggregates are mainly concentrated in this range. The particle size distribution of the C_14_DDLPB solution becomes narrower between 50 nm and 380 nm (Figure 7b), indicating that the distribution is more concentrated. The particle size distribution shown in Figure 7c is between 50 nm and 700 nm, which is a wider particle size distribution than those of other samples, indicating that the size of the formed aggregates is larger. As shown in Figure 7e, the particle size of this solution is mainly distributed between 20 nm and 80 nm, indicating that larger aggregates are not formed, which is consistent with the TEM results, and the system only forms micelles without vesicle formation.

### 2.6. Wetting Ability

**Figure 8:**
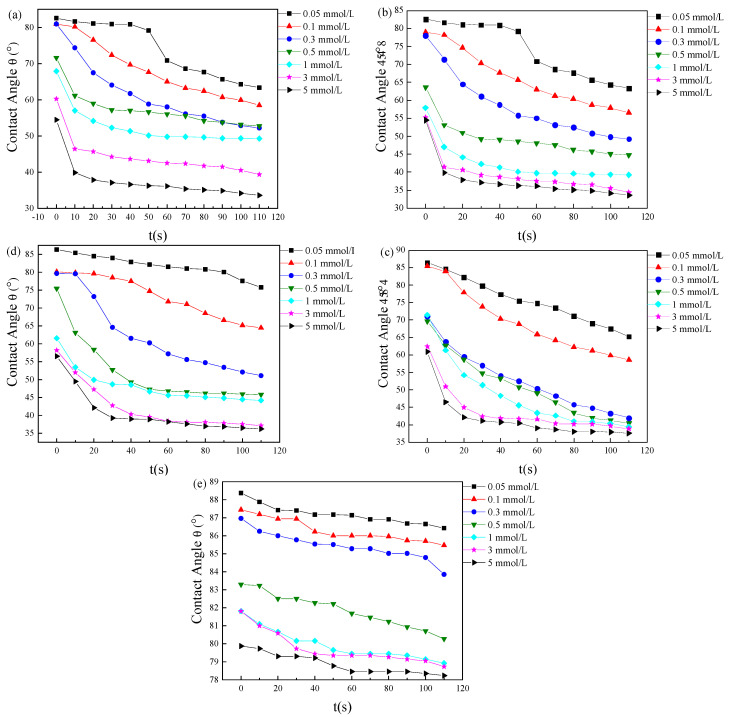
The effect of surfactant concentration on the contact angle of PTFE surface: (**a**) C_8_DDLPB, (**b**) C10DDLPB, (**c**) C12DDLPB, (**d**) C14DDLPB, and (**e**) C16DDLPB.

The changes in contact angles between different concentrations of C_n_DDLPB and PTFE over time are illustrated in Figure 8. It is evident from the graph that the contact angles of C_n_DDLPB on the surface of PTFE decrease with the increasing concentration.

In this study, C_8_DDLPB, C_10_DDLPB, C_12_DDLPB, and C_14_DDLPB have demonstrated favorable wetting properties on the PTFE surface, with contact angles reducing to 33°~38°. Particularly, C_8_DDLPB and C_10_DDLPB exhibit the ability to reduce the surface tension of water to 27.82 and 30.27 mN/m, respectively. Conversely, C_16_DDLPB shows the least wetting ability, with a contact angle only decreasing to 78° within 110 s. Its equilibrium surface tension value is measured at 59 mN/m. These findings suggest that the wetting effect of C_n_DDLPB on the PTFE surface is closely associated with its capability to lower the surface tension of water. Moreover, the impact of hydrophobic chain length in the hydrophobic group is notable [36,37].

### 2.7. Emulsifying Ability

**Figure 9:**
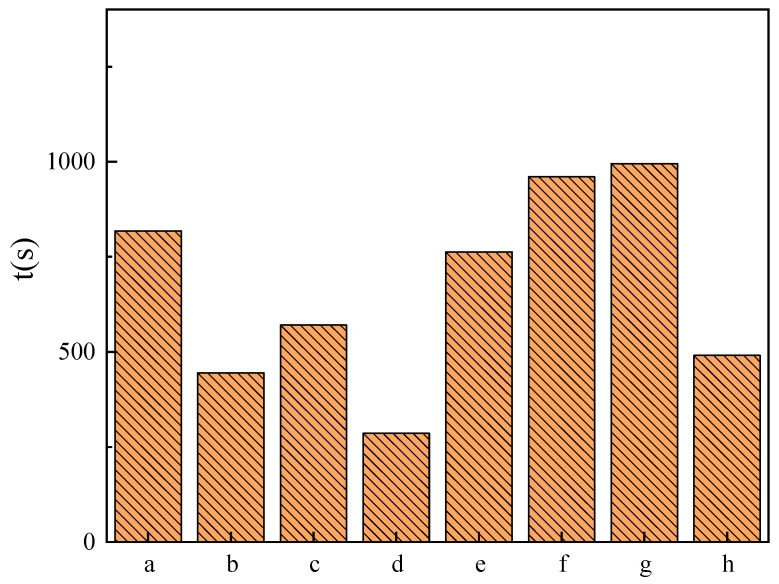
Emulsification time of soybean oil by surfactants: (**a**) 1227, (**b**) 1231, (**c**) HACC, (**d**) C_8_DDLPB, (**e**) C_10_DDLPB, (**f**) C_12_DDLPB, (**g**) C_14_DDLPB, and (**h**) C_16_DDLPB.

The emulsification capacity test results for soybean oil are shown in Figure 9. As the carbon chain length increases, the emulsification capacity initially increases and then decreases. Specifically, C_14_DDLPB exhibits the best emulsification performance with an emulsification time of 16.6 min, while the emulsification effect is poorest with a carbon chain length of eight. Furthermore, when the hydrophobic chain length becomes too long, as in the case of C_16_DDLPB, the emulsification ability also weakens. This trend parallels that observed for the single-chain glucose acylamide quaternary ammonium salt C_n_DGMAPB synthesized by our research group [38]. The hydrophobic chain length of C_n_DDLPB significantly influences the emulsification capacity of soybean oil. Poor emulsification performance is observed with hydrophobic chain lengths of 8 and 16, whereas a moderate hydrophobic chain length demonstrates good emulsification.

### 2.8. Foam Properties

**Figure 10:**
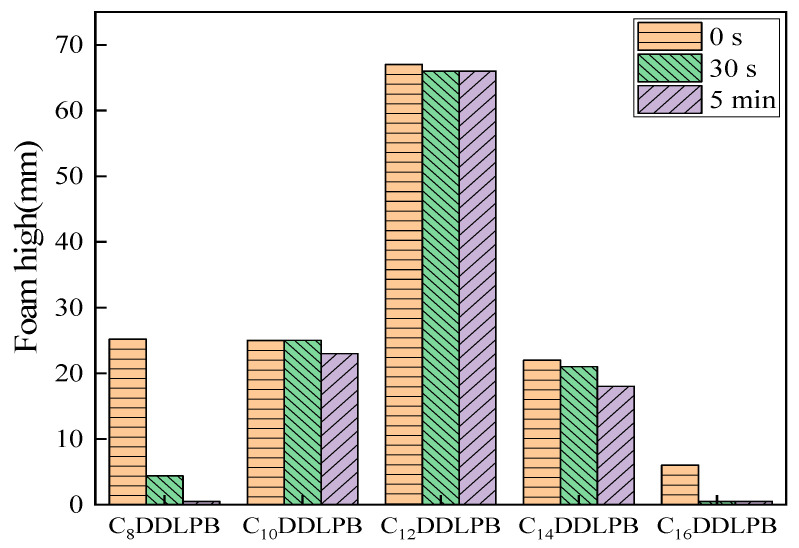
Graph of the foam properties of C_n_DDLPB surfactants.

The foam performance of C_n_DDLPB surfactants in deionized water was evaluated using the Ross–Miles method, and the results are shown in Figure 10. From the graph, it is evident that C_12_DDLPB surfactant exhibits superior foamability, with a foam height reaching 67 mm. After 30 s, the foam height decreases by only 1 mm, and there is no further change in foam height over the subsequent 5 min, indicating excellent foam stability. However, compared to common anionic surfactants (foam height of >100 mm), it belongs to the category of low-foaming surfactants. C_8_DDLPB, C_10_DDLPB, and C_14_DDLPB initially exhibit similar foam heights of 25 mm, 25 mm, and 22 mm, respectively, indicating relatively poor foamability. On the other hand, the foam generated by C_8_DDLPB and C_16_DDLPB dissipates rapidly, classifying them as low-foaming surfactants overall. These surfactants hold promise for applications in low-foam detergents and related products.

### 2.9. Antistatic Performance

The surface resistivity of polyester fabric before and after treatment was measured using a high-resistance meter, with the effectiveness of the surfactant’s antistatic properties evaluated based on the magnitude of the decrease in surface resistivity logarithm values (△lgρ_s_). A higher △lgρ_s_ value indicates better antistatic performance. A series of synthesized lactose-based quaternary ammonium salt surfactants and commercially available antistatic agent, dihydroxyethyl stearylamine nitrate (SN), were tested, and the experimental results are presented in Table 2: at a concentration of 0.35 g/L, SN > C_8_DDLPB > C_10_DDLPB > C_12_DDLPB > C_14_DDLPB > C_16_DDLPB, with C_8_DDLPB exhibiting the largest △lgρ_s_ among the C_n_DDLPB surfactants, thereby indicating the best antistatic effect. The antistatic effects of C_8_DDLPB and C_10_DDLPB are approaching those of SN. Additionally, there is a trend of decreasing antistatic performance with increasing carbon chain length in C_n_DDLPB.

From Table 2, it can be observed that the maximum adsorption (Г_max_) of C_n_DDLPB decreases with the increase in carbon chain length. Among the synthesized surfactants, C_8_DDLPB and C_10_DDLPB exhibit the highest Г_max_ values, which are 1.890 × 10^−10^ mol·cm^−2^ and 1.675 × 10^−10^ mol·cm^−2^, respectively. These results suggest that their antistatic effects may be related to their adsorption on the fabric surface.

### 2.10. Salt-Resistant Performance

This study investigated the salt resistance of DDAC and synthesized C_n_DDLPB surfactants at different concentrations in various salt environments, as shown in Table 3, Table 4 and Table 5. When the concentration of sodium chloride added was 50 g/L, C_8_DDLPB and C_14_DDLPB exhibited transmittance rates of 94.19% and 99.08%, respectively, with excellent transmittance and no precipitation, indicating good salt resistance. In the presence of 35 g/L sodium chloride in the C_12_DDLPB solution, the transmittance was 93.76%, demonstrating some degree of salt resistance.

At a concentration of 50 g/L magnesium sulfate, C_8_DDLPB and C_14_DDLPB maintained transmittance rates of 99.54% and 86.90%, respectively, indicating strong salt resistance. In the presence of NaCl, CaCl_2_, and MgSO_4_, C_8_DDLPB consistently exhibited excellent salt resistance.

In comparison with DDAC, the series of C_n_DDLPB synthesized in this study have lactose-based hydrophilic groups containing numerous hydroxyl groups with strong negative polarity. Compared to micelles, these groups can readily attract more metal cations, resulting in a significant reduction in the concentration of unbound counterions in the solution, thereby hindering the formation of precipitates to some extent.

### 2.11. Antibacterial Performance

In this study, Gram-positive bacteria (*Staphylococcus aureus*) and Gram-negative bacteria (*Escherichia coli*) were used as samples to assess the antibacterial efficacy of C_12_DDLPB and C_14_DDLPB. Each group selected an appropriate dilution gradient for antibacterial rate calculation, utilizing the following formula:

(4) $$\mathrm{Antibacterial}\;\mathrm{Rate}(\%)=\frac{C\;\text{-}\;E}{C}\times 100\%$$

where C denotes the average bacterial count of the control sample, and E denotes the average bacterial count of the experimental sample.

Quaternary ammonium salt surfactants carry a positive charge in water, allowing them to interact with negatively charged microbial surfaces, leading to adsorption and the formation of small aggregates that adhere to the cell wall. This process inhibits microbial growth. Additionally, the hydrophobic groups of these surfactants interact with the hydrophilic groups of microbial cells, altering the permeability of the cell membrane. This results in membrane damage and leakage of intracellular substances. Furthermore, the abundance of positive charges can coagulate and denature proteins within microbial cells, affecting cell metabolism and achieving disinfection and antibacterial effects [39].

From Table 6 and Table 7, it can be observed that at a concentration of 350 ppm, C_12_DDLPB exhibits excellent antibacterial performance against *Escherichia coli* and *Staphylococcus aureus*, with antibacterial rates reaching 99.29% and 95.28%, respectively. Conversely, C_14_DDLPB demonstrates poorer antibacterial efficacy, with antibacterial rates against *Escherichia coli* and *Staphylococcus aureus* at 73.20% and 76.91%, respectively. The antibacterial effect is more pronounced when the hydrophobic chain length is 12, which aligns with the previous literature [40]. Therefore, C_12_DDLPB holds promise as a novel disinfectant product for potential applications.

## 3. Experimental

### 3.1. Materials and Instruments

Lactobionic acid (99%) was purchased from Aldrich, and chemically pure octyl bromide, decyl bromide, dodecyl bromide, tetradecyl bromide, and hexadecyl bromide were purchased from Shanghai Bohua Biochemical Reagent Co. Ltd., Shanghai, China, and N,N-dimethyl dipropylenetriamine (99.15%) was purchased from Guangdong Swell River Chemical Reagent Co. Ltd., Wengjiang, China. Deuterated dimethyl sulfoxide [DMSO] (99.9%) was purchased from Shanghai McLean Biochemical Technology Co., Shanghai, China. Didecyldimethylammonium chloride [DDAC] (95%) was purchased from Shanghai McLean Biochemical Science and Technology Co., Shanghai, China. Dodecyldimethylbenzylammonium chloride [1227] (99%) and dodecyltrimethylammonium chloride [1231] (99%) were purchased from Shanghai McLean Biochemical Science and Technology Co., Shanghai, China.

FT-IR spectrometer, model VERTEX 70 (Bruker, Saarbrücken, Germany); nuclear magnetic resonance (NMR) instrument, model AVANCE III (Bruker, Germany); electrospray ionization mass spectrometer, model Q Exactive (Thermofisher, Waltham, MA, USA); vacuum drying oven, model DZF-0B (China Yuejin Medical Equipment Co., Ltd., Shanghai, China); automatic surface tension meter, model KRÜSS-Tensíío (KRÜSS, Germany); drop shape analyzer, model DSA25B (KRÜSS, Hamburg, Germany); cryogenic transmission electron microscopy, model Talos F200C (Thermo Fisher, Waltham, MA, USA); and dynamic light scattering instrument, model JEM-1011EX (China Baxter Instruments Co., Ltd., Dandong, China), were utilized.

### 3.2. Synthesis of the Intermediate N-(3′-dimethylaminopropyl)-lactamido-3-aminop-ropane (DDLPD)

Lactobionic acid (0.1 mol) and N,N-dimethyldipropylenetriamine (0.12 mol) were added to 200 mL of methanol, and the reaction was conducted under reflux conditions for 2 h. After completion of the reaction, the heating was stopped, and the reaction was allowed to cool. The solvent was evaporated using a rotary evaporator, and the resulting product was washed thrice with ether to remove the residual N,N-dimethyldipropylenetriamine, followed by drying under vacuum until a constant weight was obtained, affording the intermediate DDLPD.

### 3.3. Synthesis of N-[N’[3-(lactosyl amide)]propyl-N’-alkyl]propyl-N,N-dimethyl-N-alkylammonium Bromide (C_n_DDLPB)

First, DDLPD (0.05 mol), bromoalkane (0.15 mol), and 200 mL of anhydrous methanol were added in a 250 mL three-neck round-bottom flask equipped with a thermometer and a spherical condenser tube. Second, the reaction was performed under reflux conditions for 10 h. After completion of the reaction, the heating was stopped, and the reaction was allowed to cool. The solvent was evaporated using a rotary evaporator, and the product was washed thrice with ether and dried under vacuum until a constant weight was obtained, affording the product C_n_DDLPB.

### 3.4. Characterization of C_n_DDLPB

The samples to be tested were tested using a VERTEX 70 Fourier transform infrared (FTIR) spectral analyzer, and the samples to be tested were pressed with KBr, which was mixed homogeneously with an appropriate amount of the samples to be tested. The raw materials N,N-dimethyldipropylenetriamine, lactobionic acid, DDLPD, and C_n_DDLPB were scanned in the wavelength range of 500–4000 cm^−1^, and the structure of the products was characterized by observing the characteristic peaks of the IR spectra generated after scanning.

The synthesized products were subjected to ^1^H-NMR and ^13^C-NMR measurements (internal standard was tetramethylsilane (TMS), and solvent was deuterated dimethylsulfoxide (DMSO)) using an AVANCE III NMR spectrometer, and the molecular structure of the synthesized compounds was analyzed by NMR spectroscopy.

The synthesized compounds were detected using Q Exactive electrospray ionization mass spectrometer and identified and analyzed by ESI-MS spectra.

### 3.5. Surface Tension

The surface tension of C_n_DDLPB was determined using a completely automated surface tension meter (KRÜSS-Tensíío type) according to the hanging drop method. The prepared solutions to be tested with different concentration gradients were allowed to stand for at least 24 h before the test and then measured after a constant temperature of 20 min was achieved at 25 °C [41].

### 3.6. Dynamic Surface Tension (DST)

A DSA25B tensiometer (Krüss Company, Hamburg, Germany) was used at 25.0 ± 0.1 °C to record dynamic surface tension (DST) data. The effective surface ages were within the range of 10 ms to 200 s, with an accuracy of ±0.01 mN/m.

### 3.7. Cryogenic Transmission Electron Microscopy (Cryo-TEM)

First, an aliquot of 3.5 μL solution (3.0 mg/mL or 1.0 mg/mL) was applied to glow-discharged Quantifiol R 2/1 holey carbon grids and blotted for 3 s under a humidity of 100% at 4 °C before being plunged into liquid ethane using a Vitrobot Mark IV (Thermo Fisher Scientific, Waltham, MA, USA). The sample structure was immobilized in ice in a glassy state, and then, the sample attached to the copper mesh was transferred to a cryogenic transfer sample rod, followed by TEM imaging to observe the morphology of the 5 g/L solution of C_n_DDLPB during a stationary 2-week period [42].

### 3.8. Dynamic Light Scattering (DLS)

Reverse osmosis (RO) water was used to prepare 5 g/L of C_n_DDLPB (n = 8, 10, 12, 14, and 16) samples for measurement. The configured solution needed to be stable and homogeneous, and the temperature was set to 25 °C. An appropriate cuvette was selected, the angle of incident light and other parameters of the instrument were set, the solution (height of 1–1.5 cm) to be measured was added into the cuvette, and it was placed into the sample tank to start the measurement [43,44].

### 3.9. Wettability Study

According to the seated-drop method, the C_n_DDLPB solutions of various concentrations were aspirated using a micro-syringe at 25 °C and dropped onto a polytetrafluoroethylene (PTFE) film. The contact angle was measured using a contact angle meter, and photographs were taken, and the contact angle was recorded at 10 s intervals [45].

### 3.10. Emulsifying Performance

Emulsification performance analysis testing is commonly conducted using the cylinder method. In a 500 mL iodine flask, 40 mL of the test solution (1.0 g/L) and an equal volume of edible soybean oil are added. The mixture is vigorously shaken up and down five times, followed by a 1 min static period. This process is repeated five times. After completing the aforementioned steps, the mixture is quickly transferred to a 100 mL graduated cylinder, and timing begins immediately. The timing stops when 10 mL of water appears in the lower layer. The time taken for this process is recorded. This experiment is repeated five times to obtain the average value [46].

### 3.11. Foam Morphology Characterization

According to the Ross–Miles method, the foam performance of C_n_DDLPB was evaluated. The prepared solution (1 g/L) was placed in a constant-temperature water bath at (30 ± 0.5) °C for preheating for 30 min. Subsequently, the prepared solution was measured using a foam analyzer. The foam stability was determined based on the initial foam height and the subsequent change in foam height over a specified duration [47].

### 3.12. Antistatic Performance

The antistatic performance can be tested and analyzed according to GB/T 16801-2013. First, it is necessary to take the fabric finishing agent test solution to pre-treat the polyester fabric samples. Before and after the fabric samples are pre-treated, the surface resistivity is measured using a high resistance meter. The antistatic performance of the fabric finishing agent is evaluated based on the change in surface resistivity or its logarithmic value.

### 3.13. Salt-Resistant Performance

Prepare a 0.5 g/L solution of the surfactant. Transfer 10 mL of the solution into a test tube, followed by the addition of varying amounts of salt to create solutions of different concentrations. Allow the solutions to stand for 24 h. Subsequently, measure the transmittance of the solutions at 700 nm using a UV-Vis spectrophotometer [48].

### 3.14. Antibacterial Performance

Prepare a 35% drug stock solution using sterile water and sterilize it by filtration for subsequent use. Streak the tested *Staphylococcus aureus* and *Escherichia coli* on TSA plates and incubate them overnight at 37 °C until visible colonies appear. Select an appropriate quantity of colonies and transfer them to 5 mL of TSB liquid medium for incubation at a constant temperature of 37 °C for approximately 7 h, or until reaching an OD600 of approximately 0.6. Once the OD600 reaches 0.6 after adjustment with physiological saline, transfer the culture at a 0.1% ratio to TSB solution and incubate it at 37 °C for 18 h. Following incubation, dilute the treated bacterial suspension with PBS, and apply 100 μL of the diluted suspension onto TSA plates. Incubate the plates at 37 °C for 16 h, then photograph and count the colonies for quantification. Select an appropriate dilution gradient from each group for calculating the antibacterial rate.

## 4. Conclusions

In this study, double-chain lactobionic amide quaternary ammonium salts were synthesized by the amidation of lactobionic acid with N-N-dimethyldipropyltriamine to obtain glycosylamides, followed by quaternization with bromoalkanes of different chain lengths. The raw materials, intermediates, and target products were analyzed by Fourier transform infrared (FTIR) spectroscopy, proton nuclear magnetic resonance (^1^H NMR) spectroscopy, (^13^C NMR) spectroscopy, and electrospray ionization mass spectrometry (ESI-MS). The results indicated the successful synthesis of the target product. Through measurements including equilibrium surface tension, dynamic light scattering, and transmission electron microscopy, the surface activity, adsorption, and aggregation behavior of these compounds in aqueous solutions were investigated. Additionally, their application properties such as wetting ability, emulsification capability, foamability, antistatic performance, salt tolerance, and antibacterial activity were analyzed.

The CMC values of the five double-chain lactose amide quaternary ammonium salts (C_n_DDLPB) decreased in the order of C_8_DDLPB > C_10_DDLPB > C_12_DDLPB > C_14_DDLPB > C_16_DDLPB. With the growth of the carbon chain, the CMC of the lactose amide quaternary ammonium salts (C_n_DDLPB) decreased, and the products exhibited a good surface activity, which can reduce the surface tension of water to 27.82 mN/m. The solutions of compounds with carbon chain lengths ranging from 8 to 14 demonstrate favorable wetting and spreading properties on PTFE, with contact angles decreasing to 33°~40°.TEM images revealed that, except for C_8_DDLPB, the other products could form stable vesicle systems in an aqueous solution.

In terms of applications, C_14_DDLPB exhibits the best emulsification performance on soybean oil, with a time of 16.6 min. The foaming properties of C_n_DDLPB are generally low, characteristic of typical low-foaming products. Both C_8_DDLPB and C_10_DDLPB demonstrate excellent antistatic properties, comparable to the commonly used antistatic agent SN. C_8_DDLPB and C_14_DDLPB show good salt tolerance to NaCl, CaCl_2_, and MgSO_4_, with light transmittance exceeding 85% at a salt concentration of 50 g/L. Particularly, C_12_DDLPB displays excellent antibacterial activity against *Escherichia coli* and *Staphylococcus aureus*, with inhibition rates reaching 99.29% and 95.28%, respectively, at a concentration of 350 ppm.

Therefore, this product is a novel glucosamine-based cationic surfactant characterized by low foaming, antibacterial properties, antistatic properties, salt resistance, and the ability to form stable vesicular systems. It holds promise pertaining to applications in various fields such as drug delivery carriers, biomimetic membranes, microreactors, daily chemical industry, and food industry in the future.

## Figures and Tables

**Table 1:** Surface adsorption and aggregation parameters of C_n_DDLPB in aqueous solution.

Surfactant	CMC (mmol/L)	Γ_cmc_ (mN/m)	pC_20_	CMC/C_20_	Г_max_ (mol·cm^−2^)	A_min_ (Å^2^)	ΔG^0^_mic_ (KJ·mol^−1^)	ΔG^0^_ads_ (KJ·mol^−1^)
C_8_DDLPB	0.333	27.82	4.585	12.808	1.890 × 10^−10^	87.857	−59.96	−64.85
C_10_DDLPB	0.136	30.27	5.046	15.111	1.675 × 10^−10^	99.15	−62.75	−67.05
C_12_DDLPB	0.12	36.11	4.971	11.215	1.275 × 10^−10^	130.266	−64.66	−67.74
C_14_DDLPB	0.106	43.1	4.343	2.335	1.198 × 10^−10^	138.603	−65.33	−67.12
C_16_DDLPB	0.103	59.09	2.356	0.023	8.365 × 10^−11^	198.509	−65.28	−66.81

**Table 2:** Antistatic properties of C_n_DDLPB surfactant.

Sample Name	Concentration (g/L)	R_s_/Ω	Lg (R_s_/Ω)	Δlgρ_s_
Blank Control	0.35	2.70 × 10^−13^	13.43	0
C_8_DDLPB	0.35	2.52 × 10^−10^	10.32	3.115
C_10_DDLPB	0.35	2.29 × 10^−10^	10.36	3.075
C_12_DDLPB	0.35	4.93 × 10^−12^	12.68	0.75
C_14_DDLPB	0.35	1.99 × 10^−13^	13.2	0.235
C_16_DDLPB	0.35	2.63 × 10^−13^	13.42	0.01
SN	0.35	3.41 × 10^−10^	10.3	3.13

**Table 3:** Permeability of surfactant solutions with NaCl concentration.

Concentration (g/L)	DDAC	C_8_DDLPB	C_10_DDLPB	C_12_DDLPB	C_14_DDLPB	C_16_DDLPB
0	100%	100%	100%	100%	100%	100%
10	precipitates	96.83%	precipitates	100%	100%	90.16%
20	precipitates	96.38%	precipitates	100%	100%	69.98%
25	precipitates	95.72%	precipitates	98.86%	100%	precipitates
30	precipitates	95.72%	precipitates	98.17%	100%	precipitates
35	precipitates	95.06%	precipitates	93.76%	99.08%	precipitates
40	precipitates	95.06%	precipitates	64.57%	99.08%	precipitates
45	precipitates	94.62%	precipitates	precipitates	99.08%	precipitates
50	precipitates	94.19%	precipitates	precipitates	99.08%	precipitates

**Table 4:** Permeability of surfactant solutions with CaCl_2_ concentration.

Concentration (g/L)	DDAC	C_8_DDLPB	C_10_DDLPB	C_12_DDLPB	C_14_DDLPB	C_16_DDLPB
0	100%	100%	100%	100%	100%	100%
10	precipitates	96.83%	precipitates	100%	100%	90.16%
20	precipitates	96.38%	precipitates	100%	100%	69.98%
25	precipitates	95.72%	precipitates	98.86%	100%	precipitates
30	precipitates	95.72%	precipitates	98.17%	100%	precipitates
35	precipitates	95.06%	precipitates	93.76%	99.08%	precipitates
40	precipitates	95.06%	precipitates	64.57%	99.08%	precipitates
45	precipitates	94.62%	precipitates	precipitates	99.08%	precipitates
50	precipitates	94.19%	precipitates	precipitates	99.08%	precipitates

**Table 5:** Permeability of surfactant solutions with MgSO_4_ concentration.

Concentration (g/L)	DDAC	C_8_DDLPB	C_10_DDLPB	C_12_DDLPB	C_14_DDLPB	C_16_DDLPB
0	100%	100.00%	100.00%	100.00%	100.00%	100.00%
10	99.77%	99.54%	precipitates	precipitates	85.70%	precipitates
20	67.92%	99.54%	precipitates	precipitates	86.30%	precipitates
25	precipitates	99.54%	precipitates	precipitates	86.30%	precipitates
30	precipitates	99.54%	precipitates	precipitates	86.50%	precipitates
35	precipitates	99.54%	precipitates	precipitates	87.30%	precipitates
40	precipitates	99.54%	precipitates	precipitates	84.53%	precipitates
45	precipitates	99.54%	precipitates	precipitates	87.90%	precipitates
50	precipitates	99.54%	precipitates	precipitates	86.90%	precipitates

**Table 6:** Inhibition of *Escherichia coli* by C_12_DDLPB and C_14_DDLPB.

Sample Name	Sample Number	Dilution Factor	CFU Prorata	Average Value	Antibacterial Rate
Control Group	*Escherichia coli*1	1.00 × 10^8^	2.15 × 10^10^	2.26 × 10^10^	0
	*Escherichia coli*2	1.00 × 10^8^	2.43 × 10^10^		
	*Escherichia coli*3	1.00 × 10^8^	2.20 × 10^10^		
C_12_DDLPB	*Escherichia coli* C12-1	1.00 × 10^6^	1.47 × 10^8^	1.60 × 10^8^	99.29%
	*Escherichia coli* C12-2	1.00 × 10^6^	2.01 × 10^8^		
	*Escherichia coli* C12-3	1.00 × 10^6^	1.31 × 10^8^		
C_14_DDLPB	*Escherichia coli* C14-1	1.00 × 10^7^	4.89 × 10^9^	6.06 × 10^9^	73.20%
	*Escherichia coli* C14-2	1.00 × 10^7^	6.86 × 10^9^		
	*Escherichia coli* C14-3	1.00 × 10^7^	6.42 × 10^9^		

**Table 7:** Inhibition of *Gluconococcus aureus* by C_12_DDLPB and C_14_DDLPB.

Sample Name	Sample Number	Dilution Factor	CFU Prorata	Average Value	Antibacterial Rate
Control Group	*Staphylococcus aureus*1	1.00 × 10^7^	3.07 × 10^9^	3.06 × 10^9^	
	*Staphylococcus aureus*2	1.00 × 10^7^	3.01 × 10^9^		0
	*Staphylococcus aureus*3	1.00 × 10^7^	3.10 × 10^9^		
C_12_DDLPB	*Staphylococcus aureus* C12-1	1.00 × 10^6^	1.41 × 10^8^	1.44 × 10^8^	95.28%
	*Staphylococcus aureus* C12-2	1.00 × 10^6^	1.37 × 10^8^		
	*Staphylococcus aureus* C12-3	1.00 × 10^6^	1.55 × 10^8^		
C_14_DDLPB	*Staphylococcus aureus* C14-1	1.00 × 10^7^	5.40 × 10^8^	7.07 × 10^8^	76.91%
	*Staphylococcus aureus* C14-2	1.00 × 10^7^	7.50 × 10^8^		
	*Staphylococcus aureus* C14-3	1.00 × 10^7^	8.30 × 10^8^		

## Data Availability

Data are contained within the article and Supplementary Materials.

## Supplementary Materials

The following Supporting Information can be downloaded at: https://www.mdpi.com/article/10.3390/molecules29122749/s1, Figure S1: FT-IR spectra of (a) N-N-Dimethyldipropyltriamine, (b) Lactobionic Acid, (c) DDLPD; Figure S2: ^1^H-NMR spectra of DDLPD; Figure S3: ^1^H-NMR spectra of C_8_DDLPB ; Figure S4: ^1^H-NMR spectra of C_10_DDLPB ; Figure S5: ^1^H-NMR spectra of C_12_DDLPB ; Figure S6: ^1^H-NMR spectra of C_14_DDLPB ; Figure S7: ^1^H-NMR spectra of C_16_DDLPB; Figure S8: ^13^C-NMR spectra of DDLPD ; Figure S9: ^13^C-NMR spectra of C_8_DDLPB ; Figure S10: ^13^C-NMR spectra of C_10_DDLPB ; Figure S11: ^13^C-NMR spectra of C_12_DDLPB ; Figure S12: ^13^C-NMR spectra of C_14_DDLPB ; Figure S13: ^13^C-NMR spectra of C_16_DDLPB ; Figure S14: ESI-MS spectra of C_8_DDLPB ; Figure S15: ESI-MS spectra of C_10_DDLPB ; Figure S16: ESI-MS spectra of C_12_DDLPB ; Figure S17: ESI-MS spectra of C_14_DDLPB; Figure S18: ESI-MS spectra of C_16_DDLPB; Figure S19: cryo-EM spectra of C_n_DDLPB.
