# Supplementary material for: Aggregation Behavior and Application Properties of Novel Glycosylamide Quaternary Ammonium Salts in Aqueous Solution
Source: Molecules. 2024 Jun 9;29(12):2749. doi: 10.3390/molecules29122749 (PMC11206093; doi:10.3390/molecules29122749)
Supplement: Supplementary file 1 [file molecules-29-02749-s001.zip › molecules-3025364-supplementary.pdf]

Supporting Information

Aggregation behaviour and application properties of novel glycosylamide quaternary ammonium salts in aqueous solution

Yunkai Wang[1], Zeyu Chen[1], Erzhuang Zhang[1], Lifei Zhi[1,*], Martino Di Serio[2], Guoyong Wang[1], Yan Wang[1], Xiaoming Li[1], Xudong Liu[3], Ying Huang[4]

1. College of Chemical Engineering and Technology, Taiyuan University of Science and Technology, Taiyuan 030024, Shanxi, China
2. Department of Chemical Sciences, University of Naples Federico II, Napoli, Italy
3. Research Institute of Livestock and Aquatic Product Inspection, Shanxi Inspection and Testing Center, Shanxi, China
4. Taiyuan Hengdeyuan Animal Protection Technology Development Co., Ltd, Shanxi, China

*E-mail: lifeizhi@yeah.net

Table of Content

Characterization of C_nDDLPB (n=8, 10, 12, 14, 16)

Characterization of C_nDDLPB

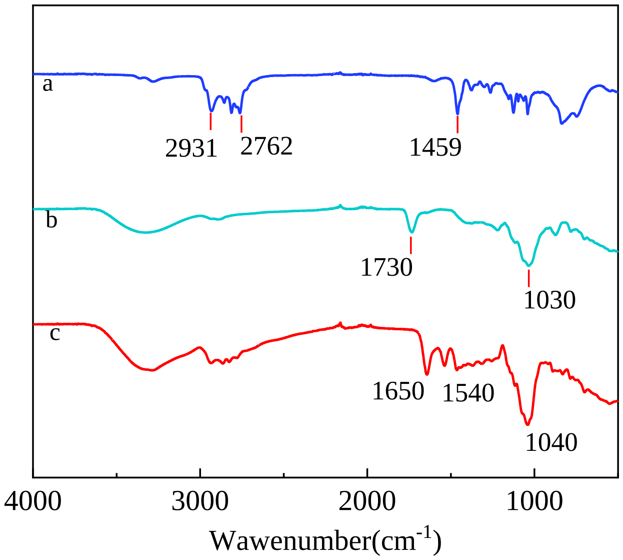

Figure S1. FT-IR spectra of (a) N-N-Dimethyldipropyltriamine, (b)Lactobionic Acid, (c) DDLPD

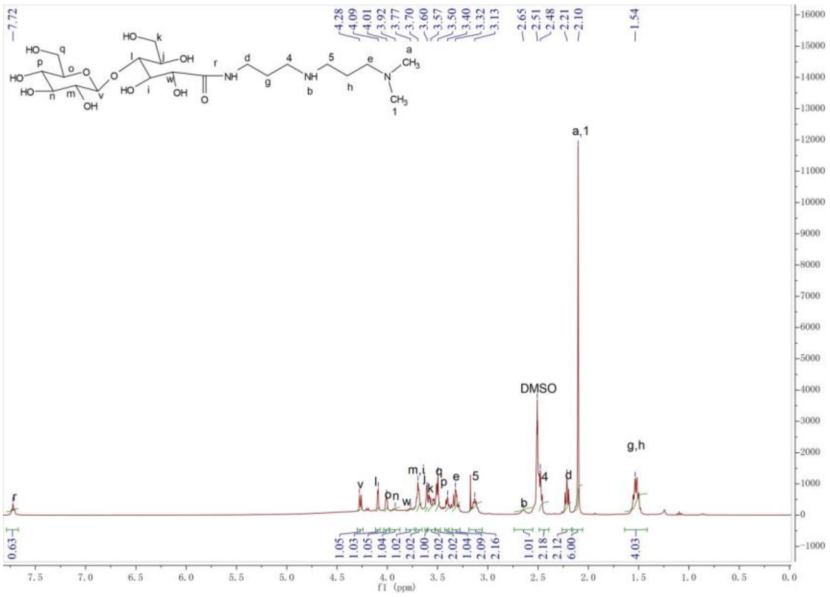

Figure S2. ^{1}H-NMR spectra of DDLPD

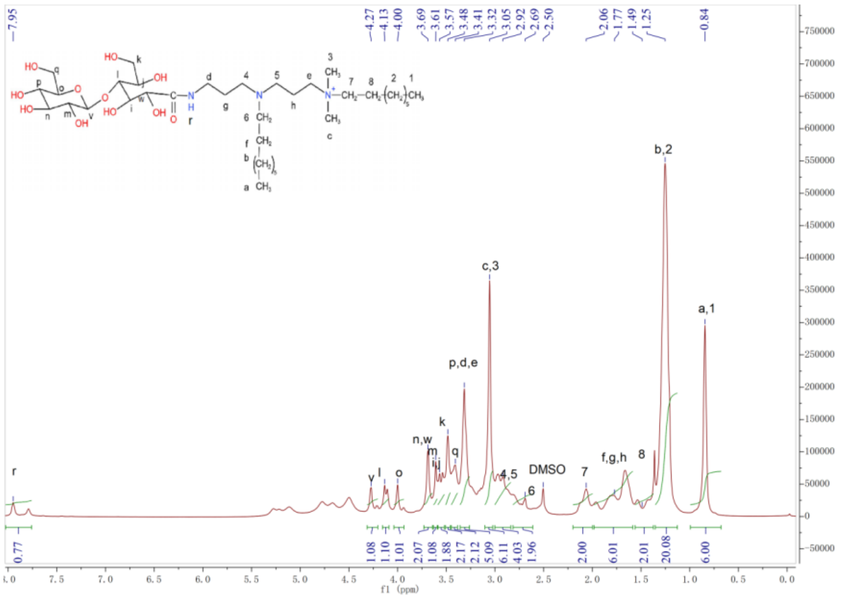

Figure S3. ^{1}H-NMR spectra of C_8DDLPB

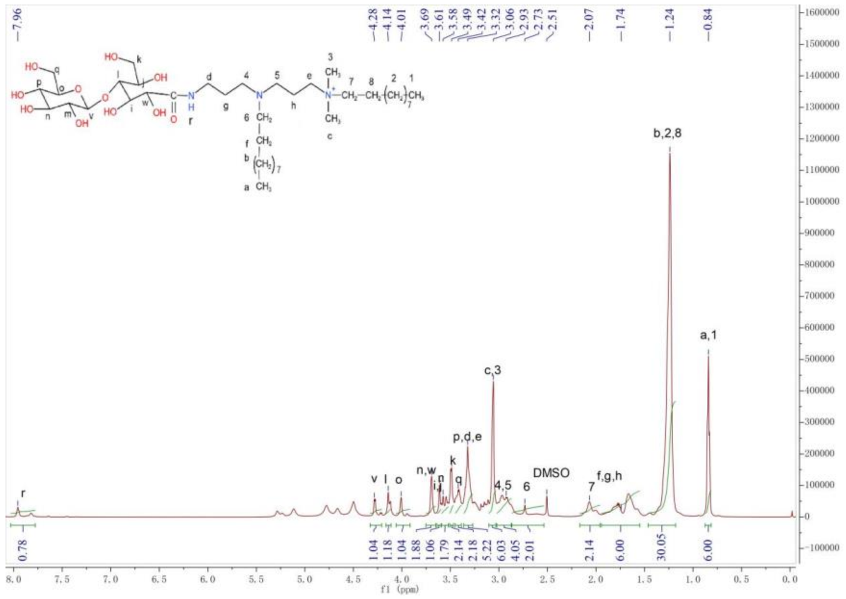

Figure S4. ^{1}H-NMR spectra of C_{10}DDLPB

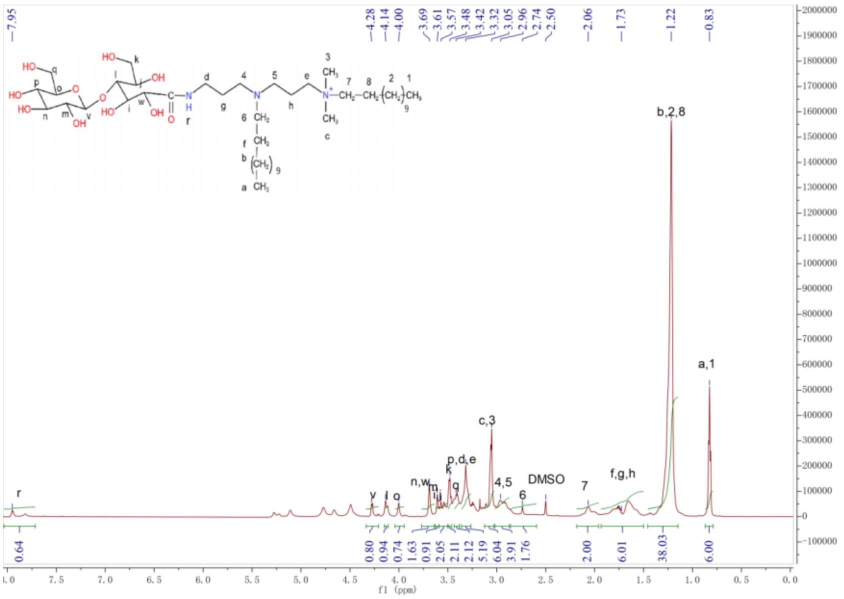

Figure S5. ^{1}H-NMR spectra of C_{12}DDLPB

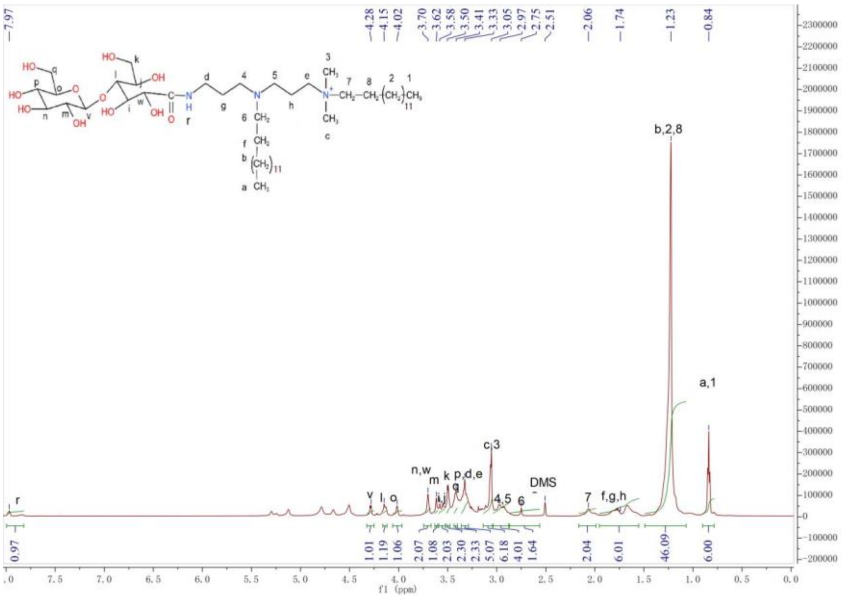

Figure S6. ^{1}H-NMR spectra of C_{14}DDLPB

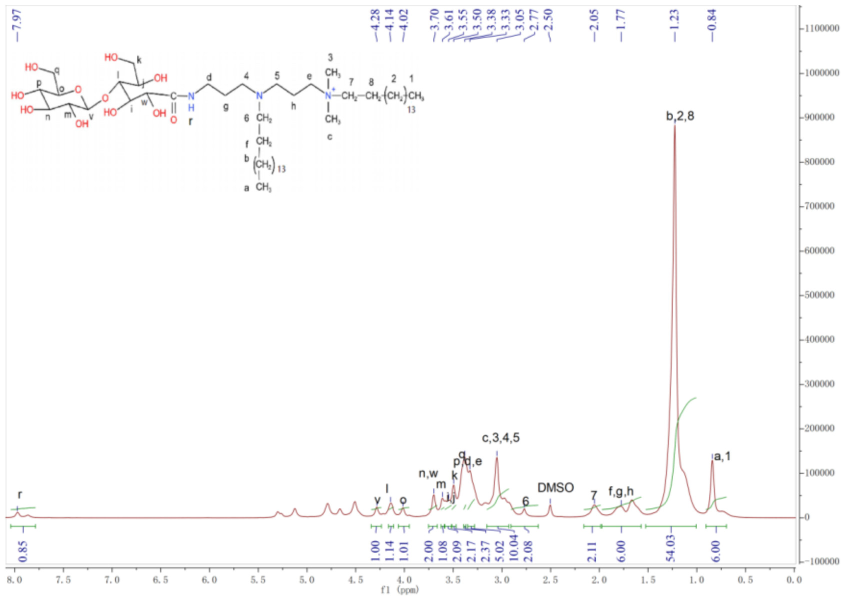

Figure S7. ^{1}H-NMR spectra of C_{16}DDLPB

DDLPD: ^{1}H-NMR(DMSO-d6, 600MHz): δ: 1.54(m, 4H, $NHCH_2C\underline{\mathbf{H_2}}$, $NHCH_2C\underline{\mathbf{H_2}}CH_2$), 2.10(s, 6H, $NC\underline{\mathbf{H_3}}C\underline{\mathbf{H_3}}$), 2.21(t, 2H, $NHCH_2CH_2C\underline{\mathbf{H_2}}NHCO$), 2.48(t, 2H, $NHC\mathbf{H_2}CH_2CH_2$ NHCO), 2.65(s, 1H, $N\underline{\mathbf{H}}$), 3.13(m, 2H, $NHC\underline{\mathbf{H_2}}CH_2CH_2N(CH_3)_2$), 3.32(m, 2H, $NHCH_2$ $CH_2C\underline{\mathbf{H_2}}N(CH_3)_2$), 3.40-4.28(m, 13H, $O\underline{\mathbf{H}}$ groups from sugar part), 7.72(s, 1H, $CON\underline{\mathbf{H}}$).

C_8DDLPB: ^{1}H-NMR(DMSO-d6, 600MHz): δ: 0.84(m, 6H, $CH_2CH_2(CH_2)_5C\underline{\mathbf{H_3}}$, $CH_2CH_2(CH_2)_5C\underline{\mathbf{H_3}}$), 1.25(s, 20H, $CH_2CH_2(C\underline{\mathbf{H_2}})_5CH_3$, $CH_2CH_2(C\underline{\mathbf{H_2}})_5CH_3$), 1.49(s, 2H, N^+ $CH_2C\underline{\mathbf{H_2}}(CH_2)_5CH_3$), 1.77(m, 6H, $NCH_2C\underline{\mathbf{H_2}}CH_2$, $NCH_2C\underline{\mathbf{H_2}}CH_2$, $NCH_2C\underline{\mathbf{H_2}}(CH_2)_5CH_3$), 2.06(m, 2H, $N^+C\underline{\mathbf{H_2}}CH_2(CH_2)_5CH_3$), 2.69(m, 2H, $NC\underline{\mathbf{H_2}}CH_2(CH_2)_5CH_3$), 2.92(m, 4H, $NC\underline{\mathbf{H_2}}$ CH_2CH_2, $NC\underline{\mathbf{H_2}}CH_2CH_2$), 3.05(d, 6H, $N^+C\underline{\mathbf{H_3}}$, $N^+C\underline{\mathbf{H_3}}$), 3.32(m, 5H, $CONHC\underline{\mathbf{H_2}}$, $N^+C\underline{\mathbf{H_2}}CH_2CH_2N$, OH groups from sugar part), 3.32-4.27(m, 12H, $O\underline{\mathbf{H}}$ groups from sugar part), 7.95(d, 1H, $CON\underline{\mathbf{H}}$).

C_{10}DDLPB : ^{1}H-NMR(DMSO-d6, 600MHz): δ: 0.84(m, 6H, $CH_2CH_2(CH_2)_7C\underline{\mathbf{H_3}}$, $CH_2CH_2(CH_2)_7C\underline{\mathbf{H_3}}$), 1.24(s, 30H, $CH_2CH_2(C\underline{\mathbf{H_2}})_7CH_3$, $N^+CH_2C\underline{\mathbf{H_2}}(C\underline{\mathbf{H_2}})_7CH_3$), 1.74(m, 6H, $NCH_2C\underline{\mathbf{H_2}}CH_2$, $NCH_2C\underline{\mathbf{H_2}}CH_2$, $NCH_2C\underline{\mathbf{H_2}}(CH_2)_7CH_3$), 2.07(m, 2H, $N^+C\underline{\mathbf{H_2}}CH_2(CH_2)_7CH_3$), 2.73(m, 2H, $NC\underline{\mathbf{H_2}}CH_2(CH_2)_7CH_3$), 2.93(m, 4H, $NC\underline{\mathbf{H_2}}CH_2CH_2$, $NC\underline{\mathbf{H_2}}CH_2CH_2$), 3.06(d, 6H, $N^+C\underline{\mathbf{H_3}}$, $N^+C\underline{\mathbf{H_3}}$), 3.32(m, 5H, $CONHC\underline{\mathbf{H_2}}$, $N^+C\underline{\mathbf{H_2}}CH_2CH_2N$, OH groups from sugar part), 3.32-4.28(m, 12H, $O\underline{\mathbf{H}}$ groups from sugar part), 7.96(d, 1H, $CON\underline{\mathbf{H}}$).

C_{12}DDLPB : ^{1}H-NMR(DMSO-d6, 600MHz): δ: 0.83(m, 6H, $CH_2CH_2(CH_2)_9\mathbf{CH_3}$, $CH_2CH_2(CH_2)_9C\underline{\mathbf{H_3}}$), 1.22(s, 38H, $CH_2CH_2(C\underline{\mathbf{H_2}})_9CH_3$, $N^+CH_2C\underline{\mathbf{H_2}}(C\underline{\mathbf{H_2}})_9CH_3$), 1.73(m, 6H, $NCH_2C\underline{\mathbf{H_2}}CH_2$, $NCH_2C\underline{\mathbf{H_2}}CH_2$, $NCH_2C\underline{\mathbf{H_2}}(CH_2)_9CH_3$), 2.06(m, 2H, $N^+C\underline{\mathbf{H_2}}CH_2(CH_2)_9CH_3$), 2.74(m, 2H, $NC\underline{\mathbf{H_2}}CH_2(CH_2)_9CH_3$), 2.96(m, 4H, $NC\underline{\mathbf{H_2}}CH_2CH_2$, $NC\underline{\mathbf{H_2}}CH_2CH_2$), 3.05(d, 6H, $N^+C\underline{\mathbf{H_3}}$, $N^+C\underline{\mathbf{H_3}}$), 3.32(m, 5H, $CONHC\underline{\mathbf{H_2}}$, $N^+C\underline{\mathbf{H_2}}CH_2CH_2N$, $O\underline{\mathbf{H}}$ groups from sugar part),3.32-4.28(m, 12H, $O\underline{\mathbf{H}}$ groups from sugar part), 7.95(d, 1H, $CON\underline{\mathbf{H}}$).

C_{14}DDLPB : ^{1}H-NMR(DMSO-d6, 600MHz): δ: 0.84(m, 6H, $CH_2CH_2(CH_2)_{11}C\underline{\mathbf{H_3}}$, CH_2 $CH_2(CH_2)_{11}C\underline{\mathbf{H_3}}$), 1.23(s, 46H, $CH_2CH_2(C\underline{\mathbf{H_2}})_{11}CH_3$, $N^+CH_2C\underline{\mathbf{H_2}}(C\underline{\mathbf{H_2}})_{11}CH_3$), 1.74(m, 6H, $NCH_2C\underline{\mathbf{H_2}}CH_2$, $NCH_2C\underline{\mathbf{H_2}}CH_2$, $NCH_2C\underline{\mathbf{H_2}}(CH_2)_{11}CH_3$), 2.06(m, 2H, $N^+C\underline{\mathbf{H_2}}CH_2(CH_2)_{11}CH_3$), 2.75(m, 2H, $NC\underline{\mathbf{H_2}}CH_2(CH_2)_{11}CH_3$), 2.97(m, 4H, $NC\underline{\mathbf{H_2}}CH_2CH_2$, $NC\underline{\mathbf{H_2}}CH_2CH_2$), 3.05(d, 6H, $N^+C\underline{\mathbf{H_3}}$, $N^+C\underline{\mathbf{H_3}}$), 3.33(m, 5H, $CONHC\underline{\mathbf{H_2}}$, $N^+C\underline{\mathbf{H_2}}CH_2CH_2N$, $O\underline{\mathbf{H}}$ groups from sugar part),3.33-4.28(m, 12H, $O\underline{\mathbf{H}}$ groups from sugar part), 7.97(d, 1H, $CON\underline{\mathbf{H}}$).

C_{16}DDLPB : ^{1}H-NMR(DMSO-d6, 600MHz): δ: 0.84(m, 6H, $CH_2CH_2(CH_2)_{13}C\underline{\mathbf{H_3}}$, $CH_2CH_2(CH_2)_{13}C\underline{\mathbf{H_3}}$), 1.23(s, 54H, $CH_2CH_2(C\underline{\mathbf{H_2)_{13}}}CH_3$, $N^+CH_2C\underline{\mathbf{H_2}}(C\underline{\mathbf{H_2)_{13}}}CH_3$), 1.77(m, 6H, $NCH_2C\underline{\mathbf{H_2}}CH_2$, $NCH_2C\underline{\mathbf{H_2}}CH_2$, $NCH_2C\underline{\mathbf{H_2}}(CH_2)_{13}CH_3$), 2.05(m, 2H, $N^+C\underline{\mathbf{H_2}}CH_2(CH_2)_{13}CH_3$), 2.77(m, 2H, $NC\underline{\mathbf{H_2}}CH_2(CH_2)_{13}CH_3$), 3.05(m, 10H, $NC\underline{\mathbf{H_2}}CH_2CH_2$, $NC\underline{\mathbf{H_2}}CH_2CH_2$, $N^+C\underline{\mathbf{H_3}}$, $N^+C\underline{\mathbf{H_3}}$), 3.33(m, 5H, $CONHC\underline{\mathbf{H_2}}$, $N^+C\underline{\mathbf{H_2}}CH_2CH_2N$, OH groups from sugar part),3.33-4.28(m, 12H, O**<u>H</u>** groups from sugar part), 7.97(d, 1H, CON**<u>H</u>**).

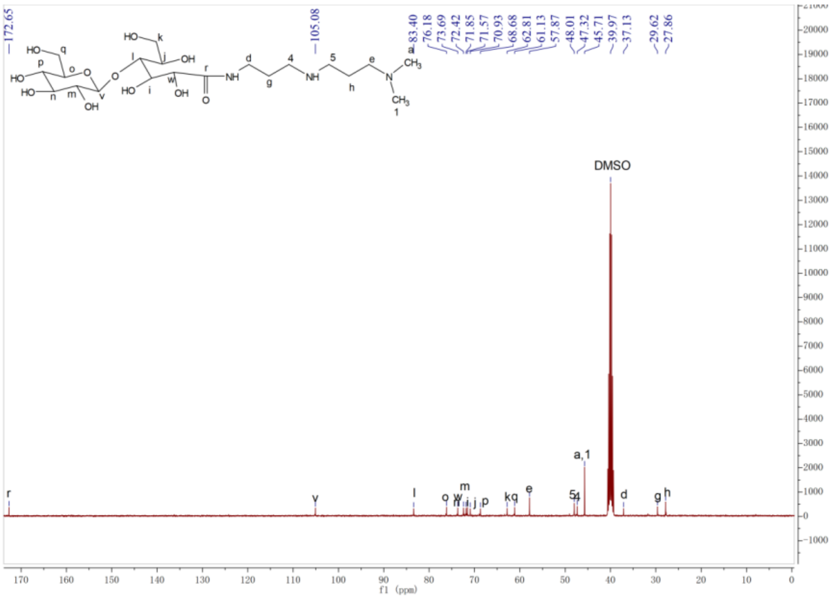

Figure S8. ^{13}C-NMR spectra of DDLPD

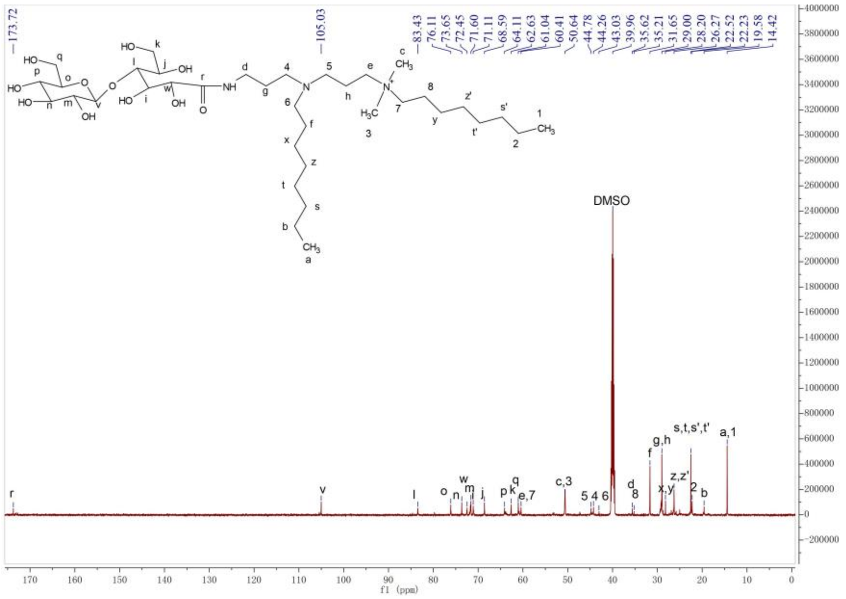

Figure S9. ^{13}C-NMR spectra of C_8DDLPB

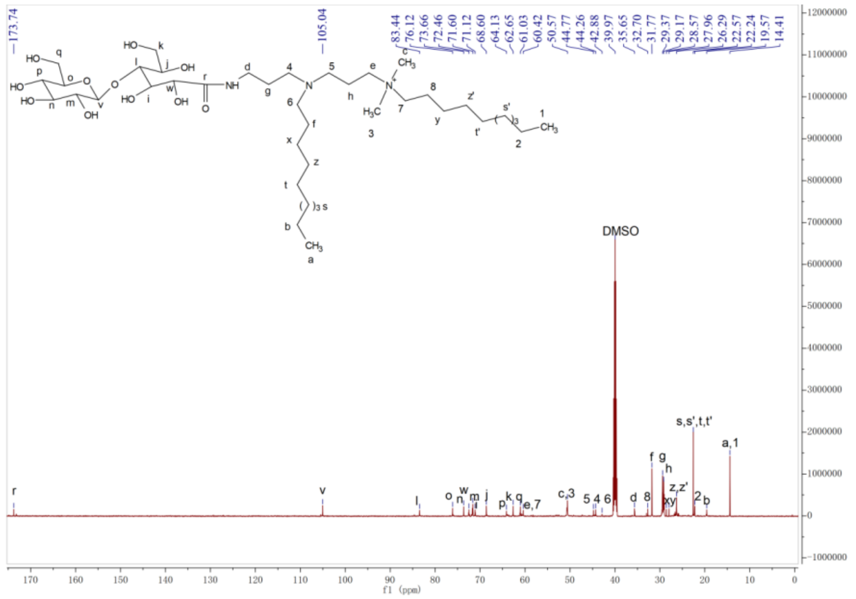

Figure S10. ^{13}C-NMR spectra of C_{10}DDLPB

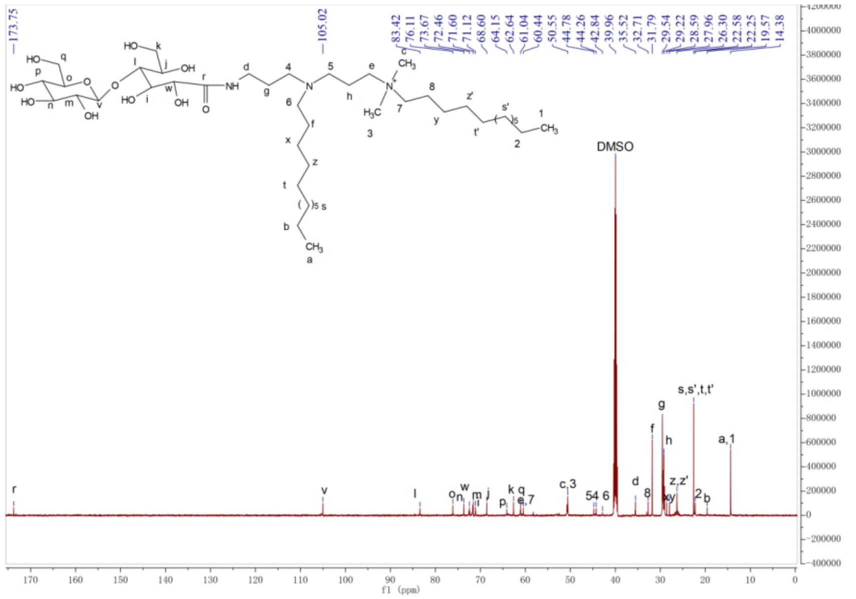

Figure S11. ^{13}C-NMR spectra of C_{12}DDLPB

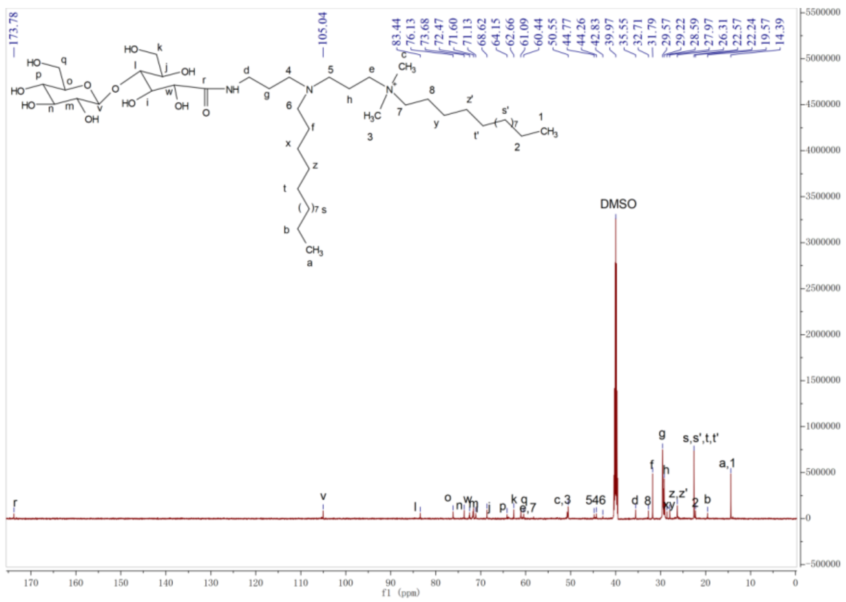

Figure S12. ^{13}C-NMR spectra of C_{14}DDLPB

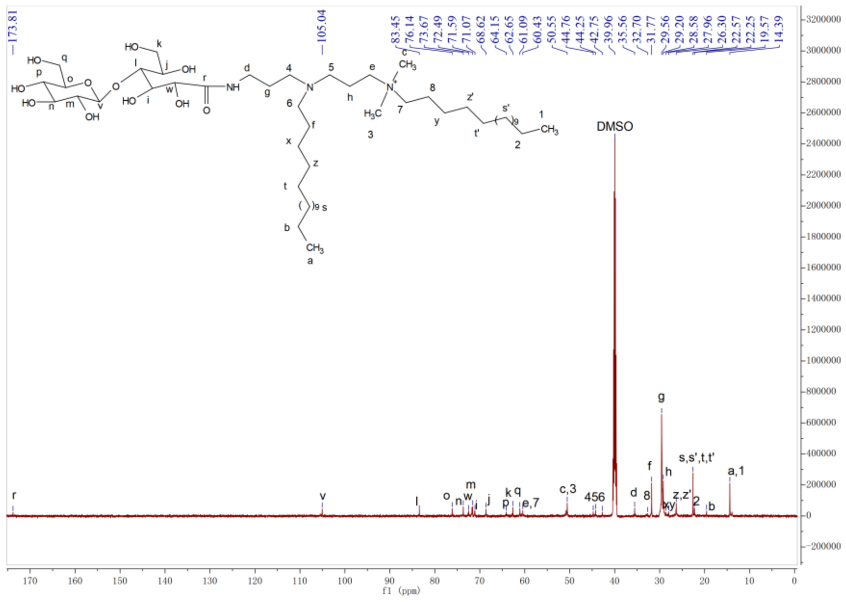

Figure S13. ^{13}C-NMR spectra of C_{16}DDLPB

DDLPD : ^{13}C-NMR(DMSO-d6, 600MHz): δ: 27.86 ($NHCH_2\underline{\mathbf{C}}H_2CH_2N$), 29.62 ($CONHCH_2\underline{\mathbf{C}}H_2CH_2NH$), 37.13 ($CONH\underline{\mathbf{C}}H_2CH_2CH_2NH$), 45.71 ($N\underline{\mathbf{C}}H_3$, $N\underline{\mathbf{C}}H_3$), 47.32 ($CONHCH_2CH_2\underline{\mathbf{C}}H_2NH$), 48.01($NH\underline{\mathbf{C}}H_2CH_2CH_2N$), 57.87 ($NHCH_2CH_2\underline{\mathbf{C}}H_2N$), 61.13 ($\underline{\mathbf{C}}H_2$ OH), 62.81 ($\underline{\mathbf{C}}H_2OH$), 68.68 ($\underline{\mathbf{C}}HOH$), 70.93 ($\underline{\mathbf{C}}H$), 71.57 ($\underline{\mathbf{C}}HOH$), 71.85 ($\underline{\mathbf{C}}HOH$), 72.42 ($\underline{\mathbf{C}}HOH$), 73.69 ($\underline{\mathbf{C}}HOH$), 76.18 ($\underline{\mathbf{C}}HO$), 83.40 ($\underline{\mathbf{C}}HO$), 105.08 ($O\underline{\mathbf{C}}HO$), 172.65 ($\underline{\mathbf{C}}ONH$).

C_8DDLPB: ^{13}C-NMR(DMSO-d6, 600MHz): δ: 14.42 ($CH_2CH_2(CH_2)_5\underline{\mathbf{C}}H_3$, CH_2CH_2 $(CH_2)_5\underline{\mathbf{C}}H_3$), 19.58 ($N(CH_2)_6\underline{\mathbf{C}}H_2CH_3$), 22.23 ($N^+(CH_2)_6\underline{\mathbf{C}}H_2CH_3$), 22.52 ($N(CH_2)_4$ $\underline{\mathbf{C}}H_2\underline{\mathbf{C}}H_2$ CH_2CH_3, $N^+(CH_2)_4\underline{\mathbf{C}}H_2\underline{\mathbf{C}}H_2CH_2CH_3$), 26.27 ($N(CH_2)_3\underline{\mathbf{C}}H_2(CH_2)_3CH_3$, $N^+(CH_2)_3\underline{\mathbf{C}}H_2$ $(CH_2)_3$ CH_3), 28.20 ($N(CH_2)_2\underline{\mathbf{C}}H_2(CH_2)_4CH_3$, $N^+(CH_2)_2\underline{\mathbf{C}}H_2(CH_2)_4CH_3$), 29.00 (CONH $CH_2\underline{\mathbf{C}}H_2$ CH_2N, $NCH_2\underline{\mathbf{C}}H_2CH_2N^+$), 31.65 ($NCH_2\underline{\mathbf{C}}H_2(CH_2)_5CH_3$), 35.21 ($N^+CH_2\underline{\mathbf{C}}H_2(CH_2)_5$ CH_3), 35.62 ($CONH\underline{\mathbf{C}}H_2CH_2CH_2N$), 43.03 ($N\underline{\mathbf{C}}H_2(CH_2)_6CH_3$), 44.26 ($CONHCH_2CH_2\underline{\mathbf{C}}H_2N$),

44.78 ($N\underline{C}H_2CH_2CH_2N^+$), 50.64 ($N^+CH_3$, N^+CH_3), 60.41 ($NCH_2CH_2\underline{C}H_2N^+$, $N^+\underline{C}H_2(CH_2)_6CH_3$), 61.04 ($\underline{C}H_2OH$), 62.63 ($\underline{C}H_2OH$), 64.11 ($\underline{C}HOH$), 68.59 ($\underline{C}HOH$), 71.11 ($\underline{C}HOH$), 71.60 ($\underline{C}HOH$), 72.45 ($\underline{C}HOH$), 73.65 ($\underline{C}HOH$), 76.11 ($\underline{C}HO$), 83.43 ($\underline{C}HO$), 105.03 ($O\underline{C}HO$), 173.72 ($\underline{C}ONH$).

C_{10}DDLPB: ^{13}C-NMR(DMSO-d6, 600MHz): δ: 14.41 ($CH_2CH_2(CH_2)_7\underline{C}H_3$, $CH_2CH_2(CH_2)_7\underline{C}H_3$), 19.57 ($N(CH_2)_8\underline{C}H_2CH_3$), 22.24 ($N^+(CH_2)_8\underline{C}H_2CH_3$), 22.57 ($N(CH_2)_4(\underline{C}H_2)_{\underline{4}}CH_2CH_3$, $N^+(CH_2)_4(\underline{C}H_2)_{\underline{4}}CH_2CH_3$), 26.29 ($N(CH_2)_3\underline{C}H_2(CH_2)_5CH_3$, $N^+(CH_2)_3\underline{C}H_2(CH_2)_5CH_3$), 27.96 ($N^+(CH_2)_2\underline{C}H_2(CH_2)_6CH_3$,), 28.57 ($N(CH_2)_2\underline{C}H_2(CH_2)_6CH_3$), 29.17 ($NCH_2\underline{C}H_2CH_2N^+$), 29.37($CONHCH_2\underline{C}H_2CH_2N$), 31.77 ($NCH_2\underline{C}H_2(CH_2)_7CH_3$), 32.70 ($N^+CH_2\underline{C}H_2(CH_2)_7CH_3$), 35.65 ($CONH\underline{C}H_2CH_2CH_2N$), 42.88 ($N\underline{C}H_2(CH_2)_8CH_3$), 44.26 ($CONHCH_2CH_2\underline{C}H_2N$), 44.77 ($N\underline{C}H_2CH_2CH_2N^+$), 50.57 ($N^+CH_3$, N^+CH_3), 60.42 ($NCH_2CH_2\underline{C}H_2N^+$, $N^+\underline{C}H_2(CH_2)_8CH_3$), 61.03 ($\underline{C}H_2OH$), 62.65 ($\underline{C}H_2OH$), 64.13 ($\underline{C}HOH$), 68.60 ($\underline{C}HOH$), 71.12 ($\underline{C}HOH$), 71.60 ($\underline{C}HOH$), 72.46 ($\underline{C}HOH$), 73.66 ($\underline{C}HOH$), 76.12 ($\underline{C}HO$), 83.44 ($\underline{C}HO$), 105.04 ($O\underline{C}HO$), 173.74 ($\underline{C}ONH$).

C_{12}DDLPB: ^{13}C-NMR(DMSO-d6, 600MHz): δ: 14.38 ($CH_2CH_2(CH_2)_9\underline{C}H_3$, $CH_2CH_2(CH_2)_9\underline{C}H_3$), 19.57 ($N(CH_2)_{10}\underline{C}H_2CH_3$), 22.25 ($N^+(CH_2)_{10}\underline{C}H_2CH_3$), 22.58 ($N(CH_2)_4(\underline{C}H_2)_{\underline{6}}CH_2CH_3$, $N^+(CH_2)_4(\underline{C}H_2)_{\underline{6}}CH_2CH_3$), 26.30 ($N(CH_2)_3\underline{C}H_2(CH_2)_7CH_3$, $N^+(CH_2)_3\underline{C}H_2(CH_2)_7CH_3$), 27.96 ($N^+(CH_2)_2\underline{C}H_2(CH_2)_8CH_3$), 28.59 ($N(CH_2)_2\underline{C}H_2(CH_2)_8CH_3$), 29.22 ($NCH_2\underline{C}H_2CH_2N^+$), 29.54($CONHCH_2\underline{C}H_2CH_2N$), 31.79 ($NCH_2\underline{C}H_2(CH_2)_9CH_3$), 32.71 ($N^+CH_2\underline{C}H_2(CH_2)_9CH_3$), 35.52 ($CONH\underline{C}H_2CH_2CH_2N$), 42.84 ($N\underline{C}H_2(CH_2)_{10}CH_3$), 44.26 ($CONHCH_2CH_2\underline{C}H_2N$), 44.78 ($N\underline{C}H_2CH_2CH_2N^+$), 50.55 ($N^+CH_3$, N^+CH_3), 60.44 ($NCH_2CH_2\underline{C}H_2N^+$, $N^+\underline{C}H_2(CH_2)_{10}CH_3$), 61.04 ($\underline{C}H_2OH$), 62.64 ($\underline{C}H_2OH$), 64.15 ($\underline{C}HOH$), 68.60 ($\underline{C}HOH$), 71.12 ($\underline{C}HOH$), 71.60 ($\underline{C}HOH$), 72.46 ($\underline{C}HOH$), 73.67 ($\underline{C}HOH$), 76.11 ($\underline{C}HO$), 83.42 ($\underline{C}HO$), 105.02 ($O\underline{C}HO$), 173.75 ($\underline{C}ONH$).

C_{14}DDLPB: ^{13}C-NMR(DMSO-d6, 600MHz): δ: 14.39 ($CH_2CH_2(CH_2)_{11}\underline{C}H_3$, $CH_2CH_2(CH_2)_{11}\underline{C}H_3$), 19.57 ($N(CH_2)_{12}\underline{C}H_2CH_3$), 22.24 ($N^+(CH_2)_{12}\underline{C}H_2CH_3$), 22.57 ($N(CH_2)_4(\underline{C}H_2)_{\underline{8}}CH_2CH_3$, $N^+(CH_2)_4(\underline{C}H_2)_{\underline{8}}CH_2CH_3$), 26.31 ($N(CH_2)_3\underline{C}H_2(CH_2)_9CH_3$, $N^+(CH_2)_3\underline{C}H_2(CH_2)_9CH_3$), 27.97 ($N^+(CH_2)_2\underline{C}H_2(CH_2)_{10}CH_3$), 28.59 ($N(CH_2)_2\underline{C}H_2(CH_2)_{10}CH_3$), 29.22 ($NCH_2\underline{C}H_2CH_2N^+$), 29.57($CONHCH_2\underline{C}H_2CH_2N$), 31.79 ($NCH_2\underline{C}H_2(CH_2)_{11}CH_3$), 32.71 ($N^+CH_2\underline{C}H_2(CH_2)_{11}CH_3$), 35.55 ($CONH\underline{C}H_2CH_2CH_2N$), 42.83 ($N\underline{C}H_2(CH_2)_{12}CH_3$), 44.26 ($CONHCH_2CH_2\underline{C}H_2N$), 44.77 ($N\underline{C}H_2CH_2CH_2N^+$), 50.55 ($N^+CH_3$, N^+CH_3), 60.44 ($NCH_2CH_2\underline{C}H_2N^+$, $N^+\underline{C}H_2(CH_2)_{12}CH_3$), 61.09 ($\underline{C}H_2OH$), 62.66 ($\underline{C}H_2OH$), 64.15 ($\underline{C}HOH$), 68.62 ($\underline{C}HOH$), 71.13 ($\underline{C}HOH$), 71.60 ($\underline{C}HOH$), 72.47 ($\underline{C}HOH$), 73.68 ($\underline{C}HOH$), 76.13 ($\underline{C}HO$), 83.44 ($\underline{C}HO$), 105.04 ($O\underline{C}HO$), 173.78 ($\underline{C}ONH$).

C_{16}DDLPB: ^{13}C-NMR(DMSO-d6, 600MHz): δ: 14.39 ($CH_2CH_2(CH_2)_{13}\underline{C}H_3$, $CH_2CH_2(CH_2)_{13}\underline{C}H_3$), 19.57 ($N(CH_2)_{14}\underline{C}H_2CH_3$), 22.25 ($N^+(CH_2)_{14}\underline{C}H_2CH_3$), 22.57 ($N(CH_2)_4(\underline{C}H_2)_{\underline{10}}CH_2CH_3$, $N^+(CH_2)_4(\underline{C}H_2)_{\underline{10}}CH_2CH_3$), 26.30 ($N(CH_2)_3\underline{C}H_2(CH_2)_{11}CH_3$, $N^+(CH_2)_3\underline{C}H_2(CH_2)_{11}CH_3$), 27.96 ($N^+(CH_2)_2\underline{C}H_2(CH_2)_{12}CH_3$), 28.58 ($N(CH_2)_2\underline{C}H_2(CH_2)_{12}CH_3$), 29.20 ($NCH_2\underline{C}H_2CH_2N^+$), 29.56($CONHCH_2\underline{C}H_2CH_2N$), 31.77 ($NCH_2\underline{C}H_2(CH_2)_{13}CH_3$), 32.70 ($N^+CH_2\underline{C}H_2(CH_2)_{13}CH_3$), 35.56 ($CONH\underline{C}H_2CH_2CH_2N$), 42.75 ($N\underline{C}H_2(CH_2)_{14}CH_3$), 44.25 ($CONH CH_2CH_2\underline{C}H_2N$), 44.76 ($N\underline{C}H_2CH_2CH_2N^+$), 50.55 ($N^+CH_3$, N^+CH_3), 60.43 ($NCH_2CH_2\underline{C}H_2N^+$, $N^+\underline{C}H_2(CH_2)_{14}CH_3$), 61.09 ($\underline{C}H_2OH$), 62.65 ($\underline{C}H_2OH$), 64.15 ($\underline{C}HOH$), 68.62 ($\underline{C}HOH$), 71.07 ($\underline{C}HOH$), 71.59 ($\underline{C}HOH$), 72.49 ($\underline{C}HOH$), 73.67 ($\underline{C}HOH$), 76.14 ($\underline{C}HO$), 83.45 ($\underline{C}HO$), 105.04 ($O\underline{C}HO$), 173.81 ($\underline{C}ONH$).

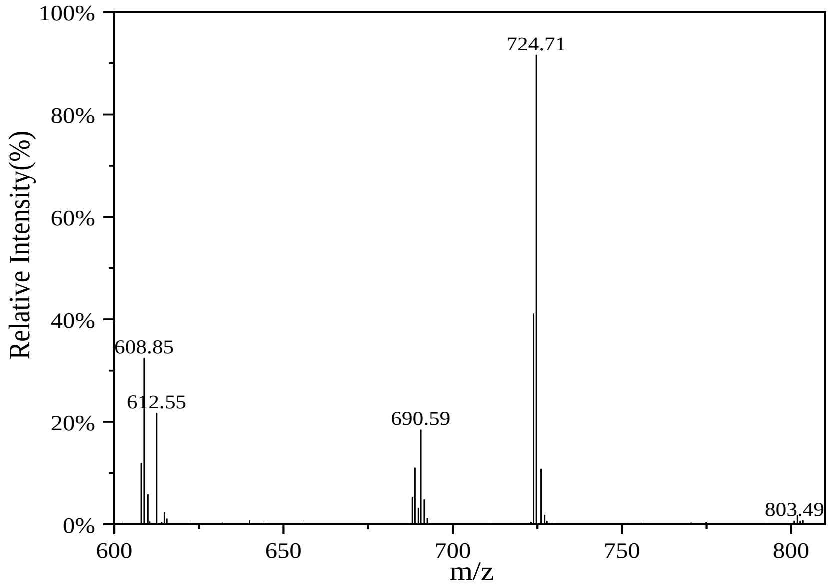

Figure S14. ESI-MS spectra of C_8DDLPB

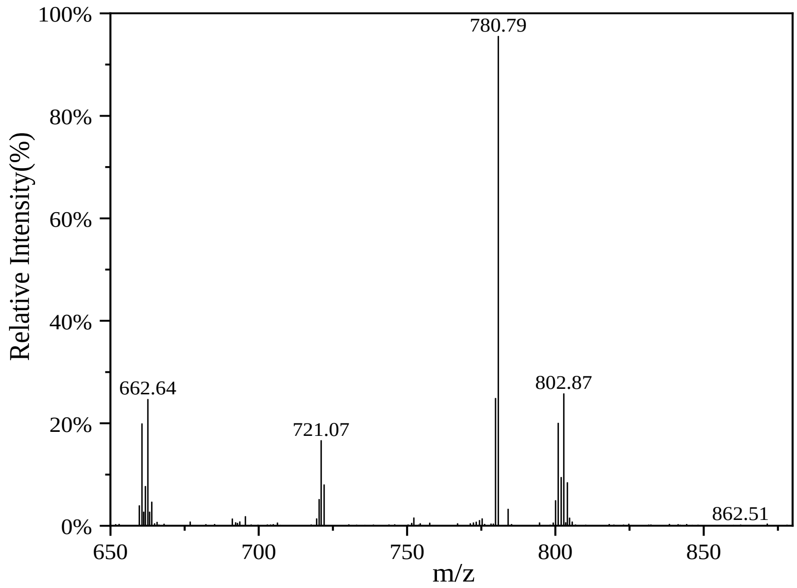

Figure S15. ESI-MS spectra of C_{10}DDLPB

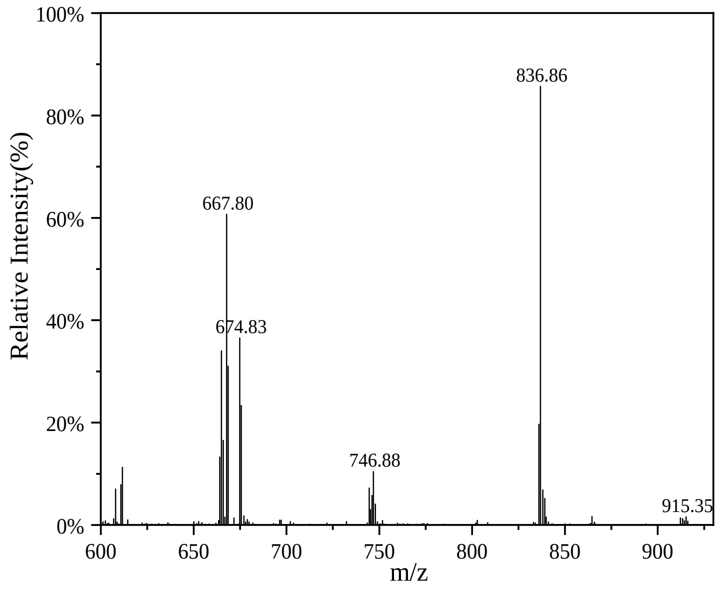

Figure S16. ESI-MS spectra of C_{12}DDLPB

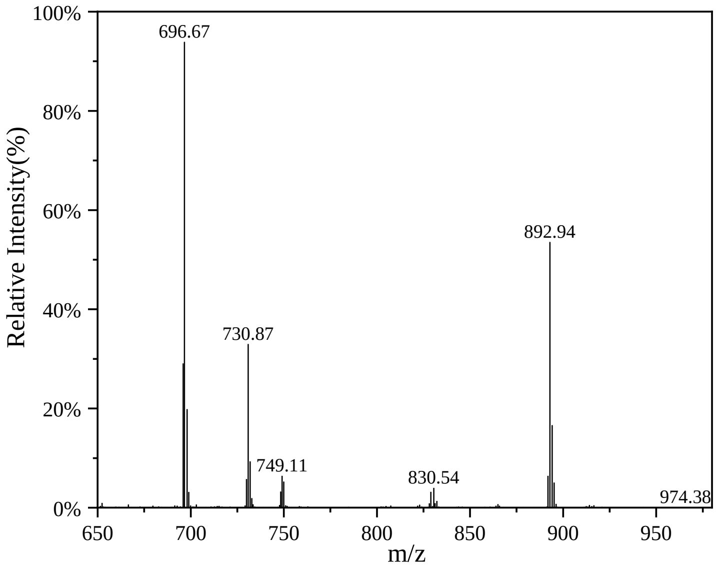

Figure S17. ESI-MS spectra of C_{14}DDLPB

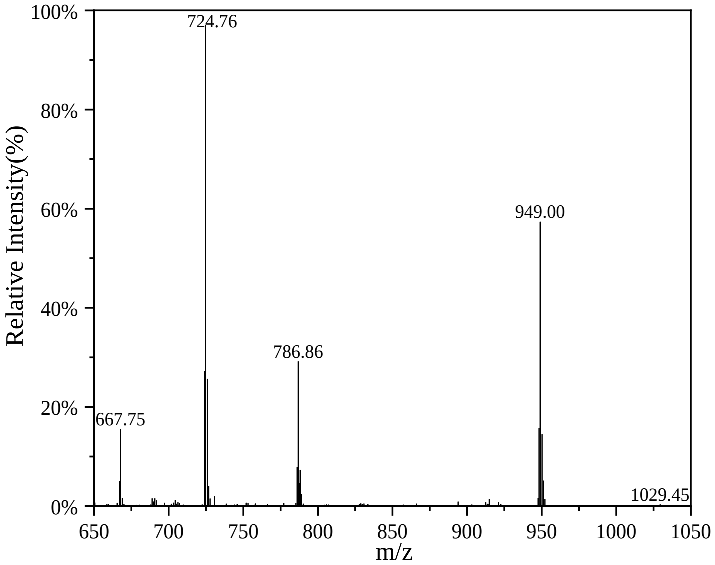

Figure S18. ESI-MS spectra of C_{16}DDLPB

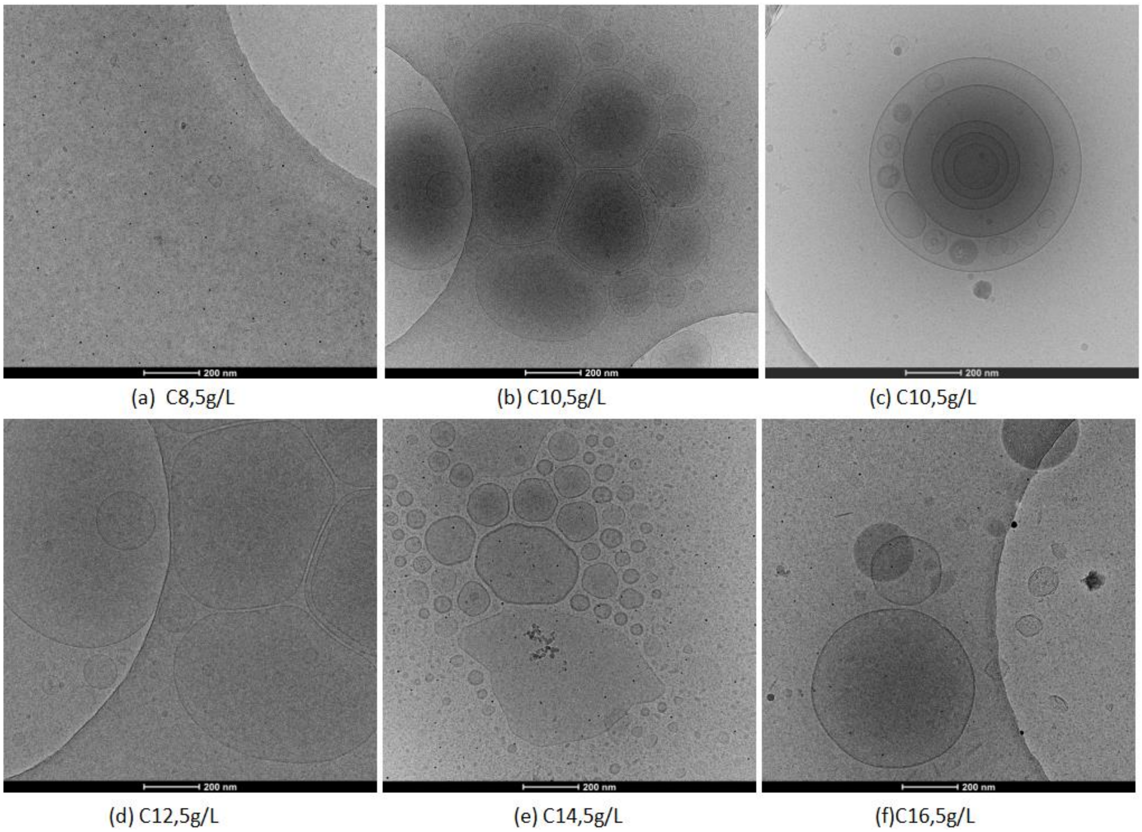

Figure S19. cryo-EM spectra of C_nDDLPB
